# Osteopetrosis-like disorders induced by osteoblast-specific retinoic acid signaling inhibition in mice

**DOI:** 10.1038/s41413-024-00353-5

**Published:** 2024-10-17

**Authors:** Siyuan Sun, Yuanqi Liu, Jiping Sun, Bingxin Zan, Yiwen Cui, Anting Jin, Hongyuan Xu, Xiangru Huang, Yanfei Zhu, Yiling Yang, Xin Gao, Tingwei Lu, Xinyu Wang, Jingyi Liu, Li Mei, Lei Shen, Qinggang Dai, Lingyong Jiang

**Affiliations:** 1 Center of Craniofacial Orthodontics, Department of Oral & Cranio-Maxillofacial Surgery, Shanghai Ninth People’s Hospital, Shanghai Jiao Tong University School of Medicine, College of Stomatology, Shanghai Jiao Tong University; National Center for Stomatology; National Clinical Research Center for Oral Disease; Shanghai Key Laboratory of Stomatology, Shanghai Research Institute of Stomatology, Shanghai, China; 2 Shanghai Institute of Immunology, Shanghai Jiao Tong University School of Medicine, Shanghai, China; 3 The 2nd Dental Center, Shanghai Ninth People’s Hospital, Shanghai Jiao Tong University School of Medicine, Shanghai, China; 4 Department of Oral Sciences, Faculty of Dentistry, University of Otago, Dunedin, 9016 New Zealand; 5 Department of Stomatology, Zhang Zhiyuan Academician Work Station, Hainan Western Central Hospital, Shanghai Ninth People’s Hospital, Danzhou, Hainan China

**Keywords:** Bone, Osteopetrosis, Bone quality and biomechanics

## Abstract

Osteopetrosis is an inherited metabolic disease, characterized by increased bone density and narrow marrow cavity. Patients with severe osteopetrosis exhibit abnormal bone brittleness, anemia, and infection complications, which commonly cause death within the first decade of life. Pathologically, osteopetrosis impairs not only the skeletal system, but also the hemopoietic and immune systems during development, while the underlying osteoimmunological mechanisms remain unclear. Osteoclastic mutations are regarded as the major causes of osteopetrosis, while osteoclast non-autonomous theories have been proposed in recent years with unclear underlying mechanisms. Retinoic acid (RA), the metabolite of Vitamin A, is an essential requirement for skeletal and hematopoietic development, through the activation of retinoic acid signaling. RA can relieve osteopetrosis symptoms in some animal models, while its effect on bone health is still controversial and the underlying mechanisms remain unclear. In this study, we constructed an osteoblast-specific inhibitory retinoic acid signaling mouse model and surprisingly found it mimicked the symptoms of osteopetrosis found in clinical cases: dwarfism, increased imperfectly-formed trabecular bone deposition with a reduced marrow cavity, thin cortical bone with a brittle skeleton, and hematopoietic and immune dysfunction. Micro-CT, the three-point bending test, and histological analysis drew a landscape of poor bone quality. Single-cell RNA sequencing (scRNA-seq) of the femur and RNA-seq of osteoblasts uncovered an atlas of pathological skeletal metabolism dysfunction in the mutant mice showing that osteogenesis was impaired in a cell-autonomous manner and osteoclastogenesis was impaired via osteoblast-osteoclast crosstalk. Moreover, scRNA-seq of bone marrow and flow cytometry of peripheral blood, spleen, and bone marrow uncovered pathology in the hematopoietic and immune systems in the mutant mice, mimicking human osteopetrosis. Results showed that hematopoietic progenitors and B lymphocyte differentiation were affected and the osteoblast-dominated cell crosstalk was impaired, which may result from transcriptional impairment of the ligands *Pdgfd* and *Sema4d*. In summary, we uncovered previously unreported pathogenesis of osteopetrosis-like disorder in mice with skeletal, hematopoietic, and immune system dysfunction, which was induced by the inhibition of retinoic acid signaling in osteoblasts, and sheds new insights into a potential treatment for osteopetrosis.

## Introduction

Osteopetrosis is an inherited metabolic disease that results from abnormal skeletal metabolism and hematopoiesis during development.^1^ According to gene mutation and age of onset, osteopetrosis is divided into various types.^2^ Among these, all osteopetrosis patients exhibit increased bone density and in severe cases, an undiscernible bone marrow cavity on radiographs.^3^ Epidemiology studies have shown that these severe patients, approximately 1 in 300 000 births,^4^ commonly die within the first decade of life.^5^ Specifically, this type of osteopetrosis, mainly exhibits three systemic disorders: craniofacial, skeletal, and hematopoietic.^6^ The craniofacial manifestations include microcephaly, short mandible, abnormal tooth eruption, and visual and auditory deficits.^7^ The skeletal manifestations include dwarfism, scoliosis, and spontaneous bone fracture.^8^ The hematopoietic manifestations include anemia, haemorrhagia, and frequent infectious complications, which are the main causes of mortality.^9^ Currently, the treatment for severe osteopetrosis is still limited to bone marrow transplantation, which results in alleviating or mildly prolonging survival but not its prevention or rehabilitation.^10^ This situation highlights the importance of further investigations into the nebulous mechanisms involved in osteopetrosis and to search for new targets for prevention and therapy.

Indeed, previous studies have focused on the pathology, and pathogenic genes involved in osteopetrosis.^10^ Autopsy has shown that the medullary cavity in osteopetrosis is filled with endochondral new bone, which leaves a very narrow space for hematopoietic cells, resulting in extramedullary hematopoiesis.^2^ Furthermore, using patient genome sequencing, several bone resorption-related mutants, including *CLCN7*, *TCIRG1*, and *TNFSF11* were identified.^11^ Accordingly, knockout murine models were constructed, in which similar poorly remodeled trabecular bone and abnormal hematopoiesis were found.^12^ Interestingly, most of these models are the result of defects in osteoclast differentiation, but comparable defects have been rarely documented in human osteopetrosis.^2^ For example, the classic op/op mice model was constructed by knockout of macrophage colony-stimulating factor (M-CSF), the essential secretory protein that induces osteoclast differentiation.^13^ As M-CSF is secreted by other cells in the bone marrow microenvironment,^14^ some researches have proposed that other cell types responsible for bone deposition and the bone microenvironment, such as osteoblasts, may play an important role in human osteopetrosis.^15^ Taken together, differing from the well-known osteoclast autonomous forms of osteopetrosis, the osteoclast non-autonomous forms, including osteoblast-originated defects, lacked related animal models and their underlying mechanisms are far from clear.

Retinoic acid (RA), the metabolite of vitamin A, is an essential nutrient in skeletal and hematopoietic development.^16^ However, the effect of RA on osteopetrosis remains controversial. In some animal models, RA relieved the osteopetrosis symptoms and was indicated as a potential therapeutic drug for the clinical treatment of osteopetrosis.^17^ A previous case reported an osteopetrosis patient who responded dramatically to therapy with the retinoid compound,^18^ while some other cases reported the failure of treatment with vitamin A for osteopetrosis.^19^ Thus, it is necessary to explore the underlying mechanism to figure out the reason. retinoic acid signaling is the key pathway for its function in vivo.^20^ Previous studies have attempted to knockout the retinoic acid receptor (RAR) family in mice to block its signaling, but they could not assess skeletal metabolism or hematopoiesis due to the compensation effect of RAR family or postnatal embryonic lethality.^21^ DnRARα mutations can inhibit the function of RAR family in vitro, which could overcome the compensation effect.^22^ Our previous study generated an osteoblast-specific dnRARα-expressing mouse model which conditionally inhibited retinoic acid signaling in osteoblasts and resulted in craniofacial skeletal deformity.^23^ Interestingly, in this study, we further explored its phenotype in long bones and presented evidence that it mimics osteopetrosis-like skeletal and hematopoietic disorders, and dwarfism with increased but imperfectly-formed trabecular bones and multiple hematopoietic disorders during postnatal development. We showed that inhibition of retinoic acid signaling in osteoblasts could impair the hematopoietic and immune systems. Thus, we showed that this model may provide new insights into the role of retinoic acid signaling in osteoblasts during skeletal, hematopoietic, and immune development with an osteoblast-dominant pathogenesis of osteopetrosis.

## Results

### Osteopetrosis-like bone was caused by inhibition of retinoic acid signaling in osteoblasts

**Fig. 1:**
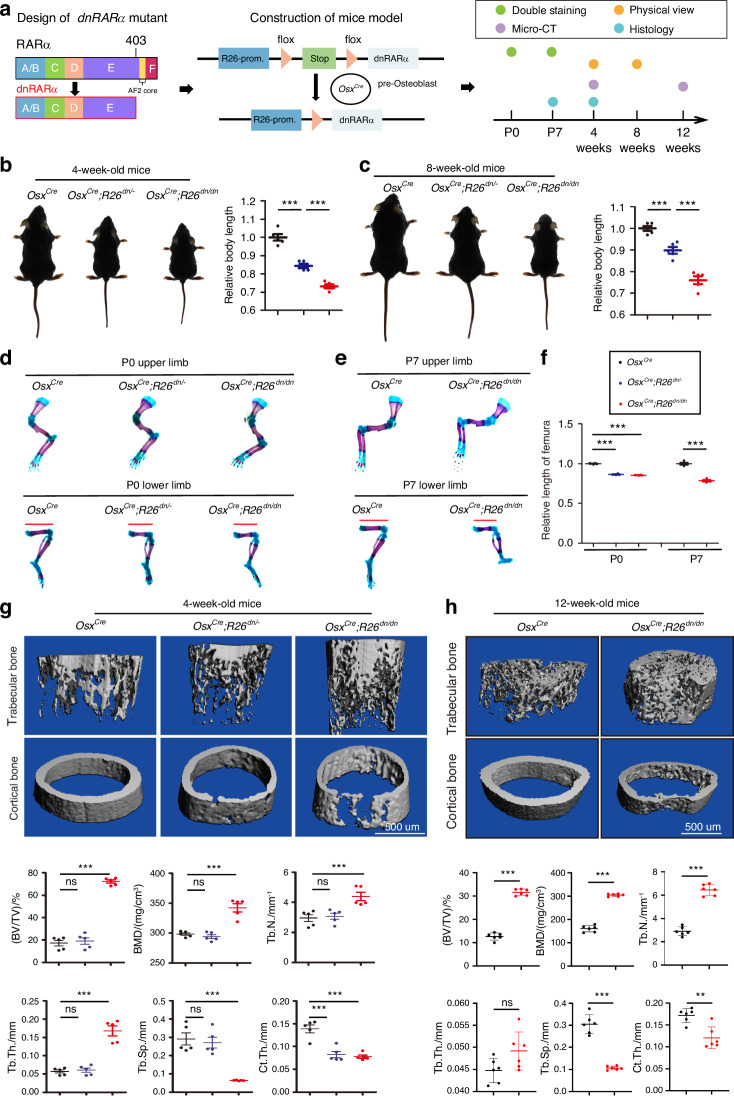
Inhibition of retinoic acid signaling in osteoblasts results in abnormally increased trabecular bone density. **a** Illustration of the *dnRARα* mutant design, mouse model construction and phenotype analysis. **b** Representative views of 4-week-old *Osx*^*Cre*^, *Osx*^*Cre*^*;R26*^*dn/-*^ and *Osx*^*Cre*^*;R26*^*dn/dn*^ mice. **c** Representative views of 8-week-old *Osx*^*Cre*^ mice, *Osx*^*Cre*^*;R26*^*dn/-*^ mice and *Osx*^*Cre*^*;R26*^*dn/dn*^ mice. **d** Upper and lower limb from *Osx*^*Cre*^, *Osx*^*Cre*^*;R26*^*dn/-*^ and *Osx*^*Cre*^*;R26*^*dn/dn*^ newborns were double-stained with alcian blue and alizarin red S. **e** Upper and lower limb from *Osx*^*Cre*^ and *Osx*^*Cre*^*;R26*^*dn/dn*^ 7-day-old mice were double-stained with alcian blue and alizarin red S. **f** Quantitative analysis of panels **d** and **e**. **g** Micro-CT images of trabecular bone and cortical bone from the femurs of 4-week-old *Osx*^*Cre*^, *Osx*^*Cre*^*;R26*^*dn/-*^ and *Osx*^*Cre*^*;R26*^*dn/dn*^ mice and their quantitative analysis, including bone volume per tissue volume (BV/TV), trabecular thickness (Tb.Th.), trabecular number (Tb.N.), trabecular space (Tb.Sp.), and cortical thickness (Ct.Th). **h** Micro-CT images of trabecular bone and cortical bone from the femurs of 12-week-old *Osx*^*Cre*^ and *Osx*^*Cre*^*;R26*^*dn/dn*^ mice and quantitative analysis. Error bars are represented as mean±S.D. ns = not significant. **P < 0.01. ***P < 0.001. Five pairs of mice were tested

The RAR family are key transcription factors involved in retinoic acid signaling,^24^ but there is no suitable model to assess their function in postnatal skeletal metabolism and hematopoiesis. Deletion of a single isoform of RAR resulted in no detectable skeletal deformity which may be due to compensation effects; whereas deletion of both RARα and RARγ resulted in severe skeletal deformity but embryonic lethality.^21^ This raised the question of how to construct a nonlethal retinoic acid signaling inhibitory model. We used a dominant-negative RARα403 mutant (*dnRARα*),^22^ which retained the function of heterodimerization, retinoid binding, and DNA binding but lacked the function of transcriptional activation^25^ (Fig. 1a). We generated an osteoblast-specific *dnRARα*-expressing mouse model (*Osx*^*Cre*^*;Rosa26*^*LSL-dnRARα/LSL-dnRARα*^, Abbrev. *Osx*^*Cre*^*;R26*^*dn/dn*^), which has been shown to be able to specifically inhibit retinoic signaling in osteoblasts in our previous study.^23^ Physical size analysis showed that 4-week-old and 8-week-old *Osx*^*Cre*^*;R26*^*dn/dn*^ mice were dwarfed relative to their control littermates (Fig. 1b, c). To determine whether the dwarfism occurred from birth, further studies at an earlier stage were performed. Shorter bone lengths of both upper and lower limbs in newborns and P7 mice were detected using alcian blue and ARS staining (Fig. 1d–f), which indicated that the skeletal disorder occurred throughout development. To further determine the skeletal phenotype, we utilized Micro-CT to analyze the skeletal characteristics of the *Osx*^*Cre*^*;R26*^*dn/dn*^ mice. Compared to controls, 4-week-old *Osx*^*Cre*^*;R26*^*dn/dn*^ mice and *Osx*^*Cre*^*;R26*^*dn/-*^ mice exhibited a reduction in cortical bone but an increase in trabecular bone mass (Fig. 1g). In detail, *Osx*^*Cre*^*;R26*^*dn/dn*^ mice showed an abnormal four-fold increased bone volume per tissue volume (BV/TV) in the femoral trabecular bone, an increase in bone mineral density (BMD), increased trabecular thickness (Tb.Th.) and trabecular number (Tb.N.) but decreased trabecular separation (Tb.Sp.) and cortical thickness (Ct.Th.) when compared with control littermates (Fig. 1g). Similarly, 12-week-old *Osx*^*Cre*^*;R26*^*dn/dn*^ mice also showed the same phenotype with an abnormal three-fold increased BV/TV when compared with control littermates (Fig. 1h). The phenotype of thin cortical bone and the increased trabecular bone density is consistent with both osteopetrosis patients and mouse models.^26^ These results suggested that the inhibition of retinoic acid signaling in osteoblasts resulted in an abnormal increase in trabecular bone density and a thin cortical bone.

**Fig. 2:**
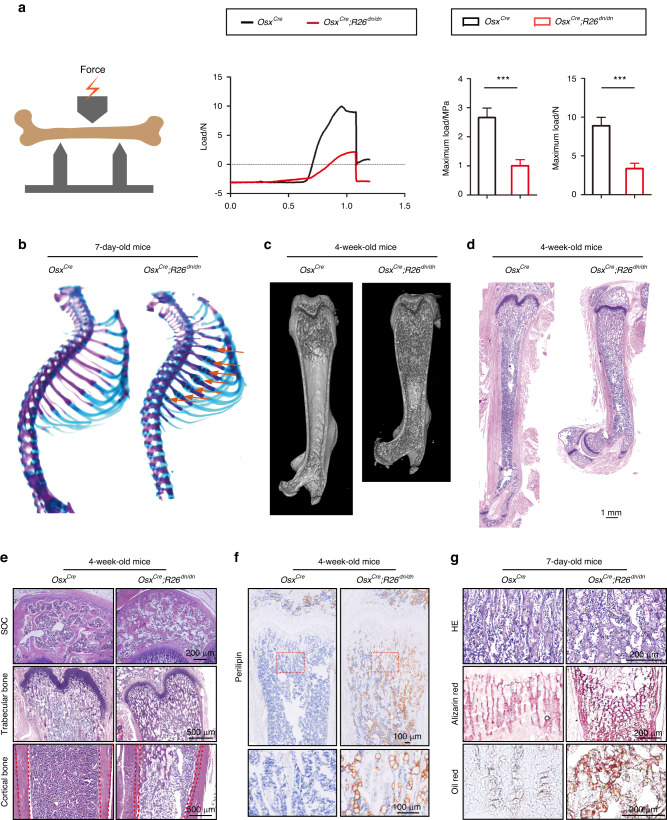
Inhibition of retinoic acid signaling in osteoblasts led to a brittle bone with narrow marrow cavity. **a** Illustration of the three-point bending test. Representative load-deflection diagram from a three-point bending test performed on femora from 4-week-old *Osx*^*Cre*^ and *Osx*^*Cre*^*;R26*^*dn/dn*^ mice. The maximum load measured during the test. *n* = 3, Error bars are represented as mean ± S.D., ***P < 0.001. **b** Ribs preparations from 7-day-old male *Osx*^*Cre*^ and *Osx*^*Cre*^*;R26*^*dn/dn*^ mice were double-stained with alcianblue and alizarin red S. Red arrows indicated pathological fracture callus. **c** Micro-CT and 3-dimensional reconstruction of femur from 4-week-old *Osx*^*Cre*^ and *Osx*^*Cre*^*;R26*^*dn/dn*^ mice. **d** H&E staining of the femurs from 4-week-old male *Osx*^*Cre*^ and *Osx*^*Cre;*^*R26*^*dn/dn*^ mice. **e** H&E staining of the SOC (upper panel), trabecular bone (middle panel) and cortical bone (lower panel) from 4-week-old male *Osx*^*Cre*^ and *Osx*^*Cre*^*;R26*^*dn/dn*^ mice. **f** Perilipin staining of the femurs from 4-week-old male *Osx*^*Cre*^ and *Osx*^*Cre;*^*R26*^*dn/dn*^ mice. **g** H&E staining, Alizarin Red staining and Oil Red staining of the trabecular bone from 7-day-old male *Osx*^*Cre*^ and *Osx*^*Cre*^*;R26*^*dn/dn*^ mice

Osteopetrosis is characterized by increased bone fragility and a high risk of bone fracture.^27^ Thus, we determined whether the abnormal increased in the trabecular bone of the osteoblastic retinoic acid signaling inhibited mice was rigid enough for physiological locomotion. The structural integrity of the femora from 4-week-old *Osx*^*Cre*^*;R26*^*dn/dn*^ mice was assessed via a three-point bending test and the results showed an obvious decrease in bone stiffness in the *Osx*^*Cre*^*;R26*^*dn/dn*^ femora when compared with controls (Fig. 2a). Notably, alcian blue and ARS staining revealed the presence of callus in most of the ribs of the 7-day-old *Osx*^*Cre*^;*R26*^*dn/dn*^ mice, suggesting frequent spontaneous fractures, which also indicated an increase in bone fragility (Fig. 2b). Furthermore, we obtained images of the femora using Micro-CT and H&E staining (Fig. 2c, d), which showed that the bone marrow cavity was filled with imperfectly formed trabecular bone in *Osx*^*Cre*^*;R26*^*dn/dn*^ mice. Specifically, histological analysis showed broadening of the distal femoral diaphysis and metaphysis, increased trabecular bone and thinner cortical bone masses, which together confirmed a weakness in the femora of *Osx*^*Cre*^*;R26*^*dn/dn*^ mice when compared to the *Osx*^*Cre*^ littermates (Fig. 2e). Moreover, both secondary ossification centers (SOC) and trabecular bone areas from 4-week-old *Osx*^*Cre*^*;R26*^*dn/dn*^ mice were filled with fat droplets (Fig. 2e), which were confirmed by perilipin immunohistochemistry staining (Fig. 2f) and might indicate an impairment of lipid metabolism. When considering the trabecular bone, we wonder if these skeletal disorders could occur at an earlier postnatal stage. The results exhibited a disruption in the regular topological arrangement of the trabecular bone, where the bone collagen fibrils appeared disordered, and fat droplets increased in the 7-day-old *Osx*^*Cre*^*;R26*^*dn/dn*^ mice, as depicted by ARS and Oil Red staining, respectively (Fig. 2g). These results indicated that retinoic acid signaling inhibition in osteoblasts resulted in brittle bone, characterized by increased imperfectly formed trabecular bone.

Moreover, we also generated a Prx1 lineage-specific *dnRARα*-expressing mouse model (*Prx1*^*Cre*^*;R26*^*dn/dn*^) (Fig. S1a). The results showed that the 4-week-old and 8-week-old *Prx1*^*Cre*^*;R26*^*dn/dn*^ mice were slightly dwarfed relative to their control littermates (Fig. S1b, c). Unexpectedly, we tried to determine the bone density and found decreased bone density and trabecular bone in 4-week-old *Prx*^*Cre*^*;R26*^*dn/dn*^ mice (Fig. S1d). In detail, it showed a decrease in BMD, decreased BV/TV in the femoral trabecular bone, decreased Tb.N. and increased Tb.Sp. compared with the control littermates (Fig. S1e). To explore the potential cause of different phenotype between *Prx1*^*Cre*^*;R26*^*dn/dn*^ mice and *Osx*^*Cre*^*;R26*^*dn/dn*^ mice, we constructed a *Prrx1Cre*^*ERT2*^*;R26-Tdtomato*^*fl/fl*^ mice for lineage tracing.^28^ The results showed the locations of *Prx1*^+^ lineage and *Osx*^+^ cells were not exactly the same (Fig. S1f, g), and we inferred that the different phenotype between *Prx1*^*Cre*^*;R26*^*dn/dn*^ mice and *Osx*^*Cre*^*;R26*^*dn/dn*^ mice may due to the difference between the two lineage cells and the difference we noticed were consistent with a previously reported research.^29^ Taken together, these demonstrated that Prx1 lineage-specific retinoic signaling inhibition could not cause osteopetrosis-like skeletal disorders.

Taken together, we constructed new osteopetrosis-like mice by inhibiting retinoic acid signaling in osteoblasts, which was characterized by increased bone density decreased bone stiffness, and a narrow marrow cavity.

### Inhibition of retinoic acid signaling in osteoblasts impaired bone anabolism and catabolism

**Fig. 3:**
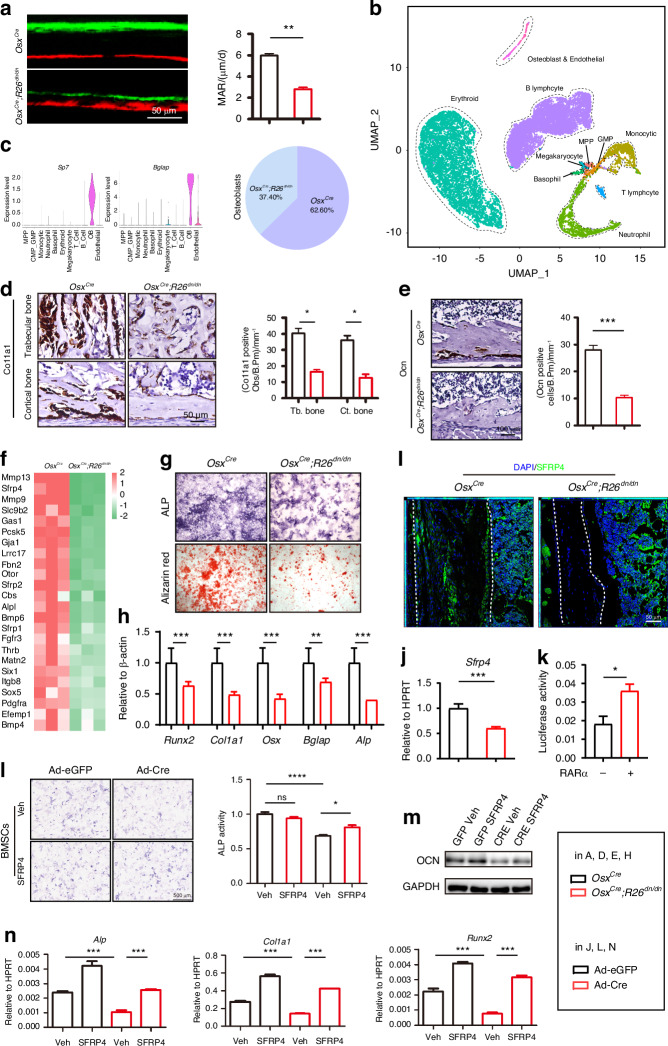
Inhibition of retinoic acid signaling in osteoblasts impairs bone anabolism. **a** Representative images of calcein-alizarin red S double labeling of the femurs from 4-week-old male *Osx*^*Cre*^ and *Osx*^*Cre*^*;R26*^*dn/dn*^ mice and quantitative parameters mineral apposition rate (MAR). **b** A total of 11 060 cells from 2-week-old *Osx*^*Cre*^ mice and a total of 10 552 cells from 2-week-old *Osx*^*Cre*^*;R26*^*dn/dn*^ mice altogether identified by scRNA-seq were visualized with UMAP. Eleven cell populations were defined and distinguished by color. Each point represents an individual cell. (**C**) Feature plots of the expression levels of marker genes for osteoblasts. Ratio of *Osx*^*Cre*^ and *Osx*^*Cre*^*;R26*^*dn/dn*^ mice-derived-osteoblasts in total osteoblasts. **d** In situ hybridization for *Col1α1* in the trabecular and cortical bone of femurs from 7-day-old male *Osx*^*Cre*^ and *Osx*^*Cre*^*;R26*^*dn/dn*^ mice and quantitation of *Col1α1*-positive cell number. **e** In situ hybridization for *Ocn* in the cortical bone of femurs from 7-day-old male *Osx*^*Cre*^ and *Osx*^*Cre*^*;R26*^*dn/dn*^ mice and quantitation of *Ocn*-positive cell number. **f** Total RNA was isolated from osteoblasts from 4-week-old male *Osx*^*Cre*^ and *Osx*^*Cre*^*;R26*^*dn/dn*^ mice, followed by RNA sequencing analysis. Heatmap analysis of osteogenesis-related genes in the *Osx*^*Cre*^ and *Osx*^*Cre*^*;R26*^*dn/dn*^ sets. **g** ALP staining and alizarin red S staining of *Osx*^*Cre*^ and *Osx*^*Cre*^*;R26*^*dn/dn*^ mouse osteoblasts after culture in osteogenic medium for 7 days and 14 days separately. **h** The relative mRNA levels of the osteogenesis-related genes *Runx2, Col1a1, Osx, Bglap*, and *Alp* in osteoblasts from *Osx*^*Cre*^ and *Osx*^*Cre*^*;R26*^*dn/dn*^ mice after culture in osteogenic medium for 7 days. **i** SFRP4 expression in the femur bone of 4-week-old *Osx*^*Cre*^ and *Osx*^*Cre*^*;R26*^*dn/dn*^ mouse by immunofluorescence staining. **j** The relative mRNA levels of *Sfrp4* in adenovirus (eGFP or Cre) infected *R26*^*dn/dn*^ osteoblasts after culture in osteogenic medium for 7 days. **k** Luciferase activity analysis for the effect of RARα on the *Sfrp4* promoter activity in the 293 T cell line (*n* = 3). **l** ALP staining of the Ad-eGFP or Ad-Cre infected and 0.1 μg/mL SFRP4 recombinant protein rescued BMSCs from *R26*^*dn/dn*^ mouse after culture in osteogenic medium for 7 days. **m** The OCN protein expression in the Ad-eGFP or Ad-Cre infected and 0.1 μg/mL SFRP4 recombinant protein rescued BMSCs from *R26*^*dn/dn*^ mouse after culture in osteogenic medium for 7 days by western blotting. **n** The mRNA expression of *Alp*, *Col1a1* and *Runx2* in the Ad-eGFP or Ad-Cre infected and 0.1 μg/mL SFRP4 recombinant protein rescued BMSCs from *R26*^*dn/dn*^ mouse after culture in osteogenic medium for 7 days by RT-qPCR Error bars are represented as mean ± SD. *P < 0.05. **P < 0.01, ***P < 0.001

Skeletal metabolism dominantly includes bone formation and bone resorption.^30^ A decrease of bone stiffness could be the result of an imbalance of osteogenesis and osteoclastogenesis in the bone microenvironment. To this end, we first assessed bone formation and found that the bone mineral apposition rate was reduced in the cortical bone of the femora in *Osx*^*Cre*^*;R26*^*dn/dn*^ mice (Fig. 3a). A number of cell lineages in the bone microenvironment regulate bone formation, and we went on to explore the key lineages that are involved in the process of retinoic acid signaling inhibition-induced osteopetrosis, as well as its underlying mechanism. We collected the femur from mice, flushed out the bone marrow, digested the bone matrix from the femora (without bone marrow) and collected the digested cells to perform scRNA-seq (3 mice for each group and both side of the femur were collected), and drew a landscape of bone matrix microenvironment from *Osx*^*Cre*^ and *Osx*^*Cre*^*;R26*^*dn/dn*^ mice (Fig. S2a). After filtering for the number of expressed genes and percentages of mitochondrial genes, a total of 11 060 cells from the *Osx*^*Cre*^ mice with a median number of RNA features of 1 478 were selected as the wt-bone group, and a total of 10 552 cells from the *Osx*^*Cre*^*;R26*^*dn/dn*^ mice with a median number of RNA features of 1 398 were selected as the ko-bone group for downstream analysis. The most significant gene expression signatures associated with each cluster were characterized (Fig. 3b). Then, unbiased clustering of scRNA-seq profiles identified 11 clusters that exhibited distinct transcriptional signatures, including B lymphocyte, erythroid, monocytic, neutrophil and osteoblasts (Fig. S2b–e). In particular, the osteogenic-related factors, such as *Sp7* and *Bglap2*, were chosen as the marker genes for osteoblasts, with a decrease of osteoblasts in the bone microenvironment was exhibited in *Osx*^*Cre*^*;R26*^*dn/dn*^ mice compared to its control littermates (Fig. 3c). Consistent with this, in-situ hybridization analysis showed that the number of collagen1α1 (Col1α1)-positive osteoblasts was reduced in the trabecular bone and cortical bone of *Osx*^*Cre*^*;R26*^*dn/dn*^ mice, as well as the number of *Ocn*-positive osteoblasts in the cortical bone of *Osx*^*Cre*^*;R26*^*dn/dn*^ mice (Fig. 3d, e). Furthermore, we tried to determine the underlying mechanism of the dysfunction of osteoblasts, and thus collected *Osx*^*Cre*^ and *Osx*^*Cre*^*;R26*^*dn/dn*^ femoral osteoblasts (BMSCs were derived from femur and osteogenic-induced for 7 days in vitro) to perform RNA-seq analysis. Overall, 1 170 genes were downregulated >2-fold and 475 genes were upregulated >2-fold in the *Osx*^*Cre*^*;R26*^*dn/dn*^ set when compared with the controls (Fig. 3f, Fig. S3a). We screened these significantly downregulated genes to perform gene ontology (GO) analysis and the results showed that they were enriched in associations with skeletal system development, ossification, and bone remodeling (Fig. S3b). These data indicated that the dysfunction of the femora osteoblasts could result from impairment of osteogenesis in a cell-autonomous manner, which was consistent with our previous research on the craniofacial bone. Furthermore, three cell types, including osteoblasts induced by femoral BMSCs, osteoblasts induced by cells digested from femur bone (without bone marrow) (F_dig cells) and osteoblasts induced by cells digested from parietal bone (P_dig cells) which correspond to cortical bone, were used in verification experiments (Fig. S3d). The results exhibited a decrease of osteoblastic differentiation in the *Osx*^*Cre*^*;R26*^*dn/dn*^ group, when compared with the common femoral osteoblasts using ALP and ARS staining (Fig. 3g), as well as deregulation of the osteogenic marker genes *Runx2*, *Col1a1*, *Osx*, *Bglap* and *Alp* by RT-qPCR (Fig. 3h). We also infected *R26*^*dn/dn*^ osteoblasts with adenovirus expressing Cre-eGFP (Ad-Cre) or control eGFP (Ad-eGFP) in vitro. The osteoblasts induced by F_dig cells and P_dig cells also showed a decrease of osteoblastic differentiation in the Ad-Cre group when compared with the Ad-eGFP group using ALP staining (Fig. S3e). Furthermore, cell proliferation was detected using CCK8, and the results showed that retinoic acid signaling inhibition impaired cell proliferation in all of the three cell types (Fig. S3f). The colony formation assay also exhibited inhibited cell proliferation in the Ad-Cre group (Fig. S3g). Altogether, these results indicated that the RA signaling could affect the proliferation and differentiation of osteoblasts both in cortical bone and trabecular bone. Among the screened genes, we noticed *Sfrp4*, the human pathogenic gene for osteopetrosis,^31^ was downregulated in *Osx*^*Cre*^*;R26*^*dn/dn*^ osteoblasts. To verify this, immunofluorescence staining in vivo suggested a decreased SFRP4 expression in the cortical bone and parietal bone of *Osx*^*Cre*^*;R26*^*dn/dn*^ mice compared to their control littermates (Fig. 3i, Fig. S3h). Moreover, the inhibition of retinoic acid signaling consistently resulted in the deregulation of *Sfrp4* expression in vitro, as determined by RT-qPCR (Fig. 3J). RAR family members are classically known as transcription factors (Fig. S3c), *dnRARα* can inhibit their transcriptional effects as a dominant negative mutant. Thus, we further explored whether RAR directly binds the promoter of *Sfrp4* to regulate its expression, and the results showed it contained several potential binding sites (Fig. S3i). Consistent with this, luciferase assays also indicated that RARα showed a significantly enhanced transcriptional effect on the *Sfrp4* promoter (Fig. 3k). Sfrp4 is known as a secreted protein that sustains bone homeostasis,^31^ we used SFRP4 recombinant protein to affect the retinoic acid signaling inhibited osteoblasts and found it partially rescued the impaired osteogenic activity (Fig. 3L), which was also confirmed at the protein and mRNA level (Fig. 3m, n). Meanwhile, the rescue effect of SFRP4 on osteogenic activity was also seen in the parietal osteoblasts (Fig. S3J-K), which suggested that the reduced SFRP4 caused by retinoic acid signaling inhibition could affect osteogenesis in the cortical bone. The expression of *Axin2* and *Id2*, the classic wnt and Bmp signaling markers were also rescued by SFRP4 in vitro (Fig. S3l). In summary, we suggest that retinoic acid signaling inhibition in osteoblasts could affect bone anabolism in a cell-autonomous manner, which may be related to the reduction of *Sfrp4* expression.

**Fig. 4:**
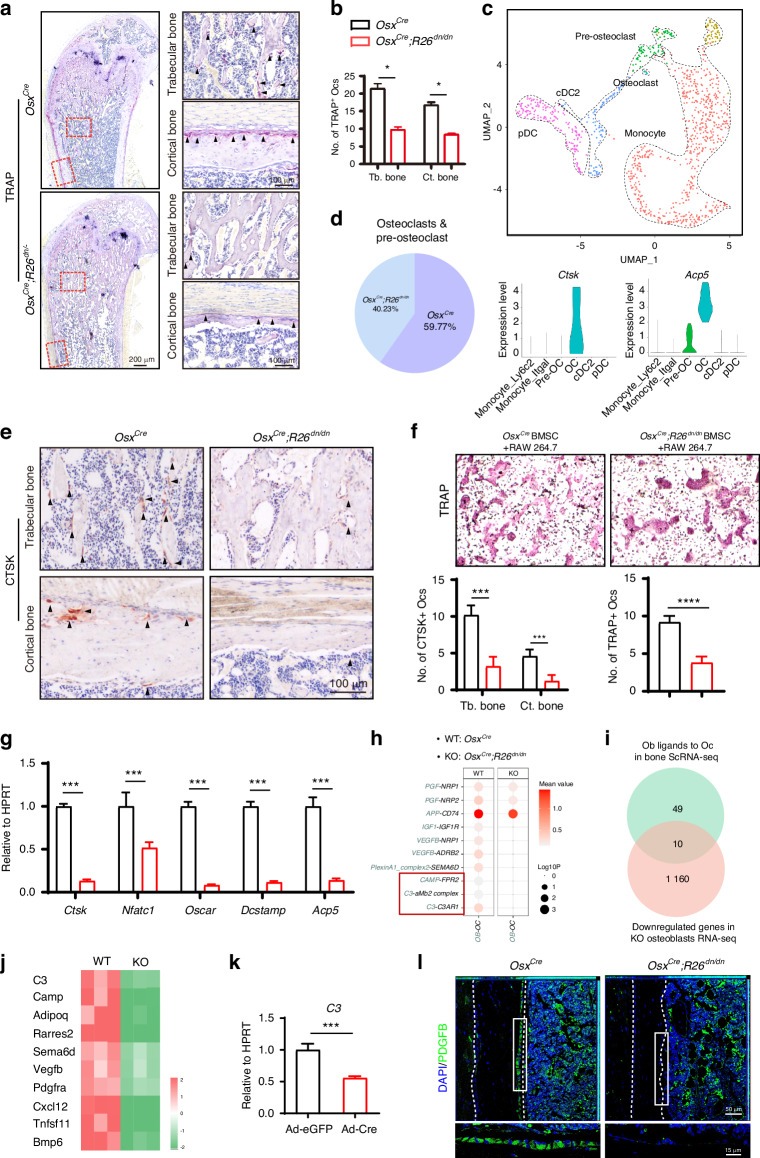
Inhibition of retinoic acid signaling in osteoblasts impairs bone catabolism. **a** TRAP staining of the trabecular and cortical bone from the femurs of 4-week-old *Osx*^*Cre*^ and *Osx*^*Cre*^*;R26*^*dn/dn*^ mice. **b** Quantitation of TRAP-positive cell number in Fig. 5a. **c** Subclustering of the “Monocytic” lineage cells from Fig. 4d, visualized as a UMAP plot. **d** Feature plots of the expression levels of marker genes from pre-osteoclasts and osteoclasts. Ratio of *Osx*^*Cre*^ and *Osx*^*Cre*^*;R26*^*dn/dn*^ mice-derived-osteoclasts and pre-osteoclasts in total osteoclasts and pre-osteoclasts. **e** CTSK immunohistochemical staining of the trabecular and cortical bone of femurs from 4-week-old *Osx*^*Cre*^ and *Osx*^*Cre*^*;R26*^*dn/dn*^ mice. **f** TRAP staining of RAW264.7 cells after coculturing with 4-week-old *Osx*^*Cre*^ and *Osx*^*Cre*^*;R26*^*dn/dn*^ mouse osteoblasts for 7 days. **g** The relative mRNA levels of osteoclast-specific genes *Ctsk, Nfatc1, Oscar, Dcstamp* and *Acp5* in RAW264.7 cells after coculturing for 7 days. **h** Cell communication analysis between osteoblasts and osteoclasts and pre-osteoclasts in *Osx*^*Cre*^ and *Osx*^*Cre*^*;R26*^*dn/dn*^ mice. **i** Number of downregulated osteoblastic ligands screened by ScRNA-seq and RNA-seq. **j** Heatmap analysis of the screened osteoblastic ligands in the *Osx*^*Cre*^ and *Osx*^*Cre*^*;R26*^*dn/dn*^ osteoblast RNA-seq sets. **k** The relative mRNA levels of the screened osteoblastic ligands *C3* in adenovirus (eGFP or Cre) infected *R26*^*dn/dn*^ osteoblasts after culture in osteogenic medium for 7 days. **l** PDGFB expression in the femoral bone of *Osx*^*Cre*^ and *Osx*^*Cre*^*;R26*^*dn/dn*^ mouse by immunofluorescence staining. Error bars are represented as mean±S.D. ns = not significant. *P < 0.05. ***P < 0.001. ****P < 0.000 1

Impairment of osteoclastic activation is an important pathological feature of osteopetrosis, as well as a potential cause of osteopetrosis-like increased bone density in our mouse model. Thus, we further focused on the change in osteoclastogenesis in *Osx*^*Cre*^*;R26*^*dn/dn*^ mice, whose severely increased bone density indicated that impaired osteoblasts may lead to abnormal osteoclastogenesis. We evaluated the bone resorption rate of the femur by analyzing tartrate-resistant acid phosphatase (TRAP)-positive osteoclasts and compared with control littermates, both trabecular and cortical bone of the femora exhibited fewer osteoclasts in the *Osx*^*Cre*^*;R26*^*dn/dn*^ mice, indicating impaired bone resorption especially in the trabecular bone (Fig. 4a, b, Fig. S4a). Osteoclasts originate from macrophages. The impairment of osteoclastogenesis could involve pre-osteoclast recruitment, osteoclast differentiation, and osteoclastic function.^32^ To this end, subclustering of the monocytic cells resulted in 5 distinct subpopulations (Fig. 4c, Fig. S4b, c). In particular, the osteoclastic differentiation factors, *Acp5* and *Ctsk*, were chosen as the marker genes for pre-osteoclasts and osteoclasts, and a decrease of osteoclasts in the bone microenvironment was exhibited in *Osx*^*Cre*^*;R26*^*dn/dn*^ mice when compared to its control littermates (Fig. 4d, Fig. S4d). Consistent with this, the immunohistochemical analysis of cathepsin K (CTSK) also showed decreased numbers of osteoclasts in both trabecular and cortical bone from 4-week-old *Osx*^*Cre*^*;R26*^*dn/dn*^ mice (Fig. 4e). These data indicated that osteoblastic disorders could affect osteoclastogenesis, and may cause an osteopetrosis-like skeletal phenotype. However, the exact mechanism remains unclear. Osteoblast–osteoclast crosstalk is one of the main patterns of skeletal metabolism, hence we wondered whether osteoblastic inhibition of retinoid signaling affected osteoclast formation via osteoblast–osteoclast interaction. Raw 264.7 cells were co-cultured with BMSCs from *Osx*^*Cre*^*;R26*^*dn/dn*^ or WT mice, and TRAP staining showed a reduction of osteoclasts in the *Osx*^*Cre*^*;R26*^*dn/dn*^ group (Fig. 4f), as well as decreased expression of the osteoclastogenic marker genes *Ctsk*, *Nfatc1*, *Oscar*, *Dcstamp* and *Acp5* (Fig. 4g), indicating impaired osteoclastic differentiation. However, osteoclastogenesis originating from the monocytic fraction of the *Osx*^*Cre*^*;R26*^*dn/dn*^ mice without osteoblast in vitro showed no significant difference when compared to its control littermates (Fig. S4e, f). Furthermore, we tried to explore the potential interactions that regulate osteoclast activity in this process and cell communication analysis between osteoblasts and osteoclasts was performed using the bone scRNA-seq data (Fig. S4g). A series of osteoclastogenesis-related interacting pairs were found to be impaired in *Osx*^*Cre*^*;R26*^*dn/dn*^ mice, which are important interactions for osteoclastic differentiation (Fig. 4h). We combined the impaired osteoblastic ligands and downregulated genes (Fig. 4i), and screened *C3*, *Camp*, and 8 other genes as being typical, which may be the potentially key factors during osteoblast-osteoclast crosstalk (Fig. 4j). The expression of typical genes were verified in the adenovirus-infected *R26*^*dn/dn*^ osteoblasts using RT-qPCR (Fig. 4k). Next, we explored and found them containing several potential binding sites (Fig. S4h). Moreover, a C3 recombinant protein was also used to interfere with Ad-cre infected *R26*^*dn/dn*^ osteoblast cocultured RAW264.7 cells and a partial rescue effect was seen (Fig. S4i-j). Here we do not eliminate other ligands that participate in osteoblast-osteoclast crosstalk that could play a role in osteoblastic retinoic acid signaling inhibition-induced osteoclastic impairment, and we identify C3 as typical. A previous study reported a similar phenotype for *Csf1*^*-/-*^ osteopetrosis mice and suggested that the decreased PDGFB resulted from the impaired osteoclastogenesis in the cortical bone might be the cause of thin cortical bone.^29^ Here we detected the expression of PDGFB in the cortical bone and found a decrease in *Osx*^*Cre*^*;R26*^*dn/dn*^ mice compared to its control littermates (Fig. 4l), which indicated that there might be a further inhibition of osteogenesis in the cortical bone caused by the impaired osteoclastogenesis in *Osx*^*Cre*^*;R26*^*dn/dn*^ mice. Collectively, these data indicated inhibition of retinoic acid signaling in osteoblasts impaired bone catabolism via osteoblast–osteoclast crosstalk, which may eventually cause an osteopetrosis-like increase in bone density.

### Disorders of hematopoiesis were caused by the inhibition of retinoic acid signaling in osteoblasts through the prevention of hematopoietic progenitor cell differentiation

**Fig. 5:**
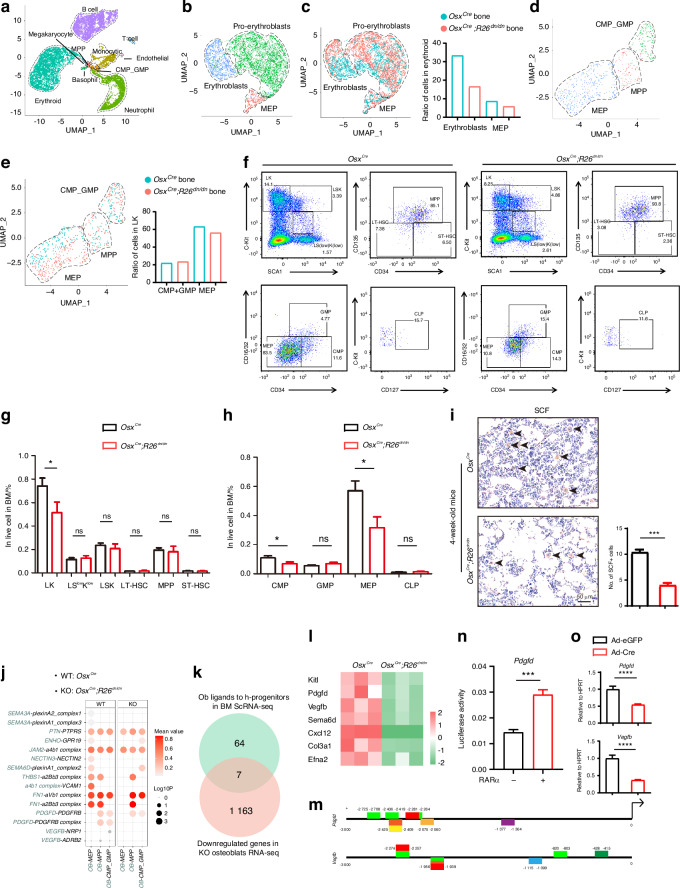
Disorders of hematopoiesis were caused by inhibition of retinoic acid signaling in osteoblasts through impairing hematopoietic progenitors differentiation. **a** A total of 6 827 cells from 2-week-old *Osx*^*Cre*^ mice and a total of 9 151 cells from 2-week-old *Osx*^*Cre*^*;R26*^*dn/dn*^ mice were identified by scRNA-seq and visualized with UMAP. Eleven cell populations were defined and distinguished by color. Each point represents an individual cell. **b** Subclustering of the “Erythroid” lineage cells from Fig. 6a visualized as a UMAP plot. **c** The ratio of MEP in the “Erythroid” lineage cells from *Osx*^*Cre*^ and *Osx*^*Cre*^*;R26*^*dn/dn*^ mice. **d** Population of hematopoietic progenitors visualized as a UMAP plot after merging the MEP, MPP and CMP_GMP subclusters. **e** The ratio of CMP_GMP and MEP in the hematopoietic progenitors from *Osx*^*Cre*^ and *Osx*^*Cre*^*;R26*^*dn/dn*^ mice, correspondingly. **f**–**h** The frequencies of LK, LS^low^K^low^, LSK, LT-HSC, MPP, ST-HSC, CMP, GMP, MEP and CLP cells in bone marrow from *Osx*^*Cre*^ and *Osx*^*Cre*^*;R26*^*dn/dn*^ mice (*n* = 4 for each group). **i** SCF immunohistochemical staining of the bone marrow of femurs from 4-week-old *Osx*^*Cre*^ and *Osx*^*Cre*^*;R26*^*dn/dn*^ mice and quantitation of SCF-positive cell number. **j** Cell communication analysis between osteoblasts and hematopoietic progenitors in *Osx*^*Cre*^ and *Osx*^*Cre*^*;R26*^*dn/dn*^ mice. **k** Number of downregulated osteoblastic ligands screened by ScRNA-seq and RNA-seq. **l** Heatmap analysis of the screened osteoblastic ligands related to hematopoietic progenitor differentiation in the *Osx*^*Cre*^ and *Osx*^*Cre*^*;R26*^*dn/dn*^ osteoblasts RNA-seq sets. **m** Predicted RAR family binding sites on the screened osteoblastic ligands *Pdgfd* and *Vegfb* promoters. **n** Luciferase activity analysis of the effect of RARα on *Pdgfd* promoter activity in the 293 T cell line (*n* = 3). **o** The relative mRNA levels of the screened osteoblastic ligands *Pdgfd* and *Vegfb* in adenovirus (eGFP or Cre) infected *R26*^*dn/dn*^ osteoblasts after culture in osteogenic medium for 7 days. Error bars are represented as mean±S.D. ns = not significant. *P < 0.05. **P < 0.01. ****P < 0.000 1

Generally, we found the change in bone phenotype and skeletal metabolism of the *Osx*^*Cre*^*;R26*^*dn/dn*^ mice to be very similar to osteopetrosis. Considering that osteopetrosis patients commonly suffer anemia, haemorrhagia, and frequent infectious complications,^33^ we wondered whether retinoic acid signaling inhibition in osteoblast lineages, could also cause hematopoietic disorders. We flushed out bone marrow and lysed the erythrocytes, then the cells in the bone marrow were collected and subjected to scRNA-seq and a single-cell atlas of the bone marrow was constructed (Fig. S2a). After filtering for the number of expressed genes and percentages of mitochondrial genes, a total of 6 827 cells from *Osx*^*Cre*^ mice with a median number of RNA features of 1 621 were selected as the wt-bone-marrow group, and a total of 9 151 cells from the *Osx*^*Cre*^*;R26*^*dn/dn*^ mice with a median number of RNA features of 1 798 were selected as the ko-bone-marrow group, for downstream analysis. Unbiased clustering of scRNA-seq profiles identified 11 clusters that exhibited distinct transcriptional signatures, including B lymphocytes, erythroid cells, monocytic cells, neutrophils and others (Fig. 5a, Fig. S5). Further focusing on the anemic feature of osteopetrosis patients, subclustering on the erythroid cells resulted in three distinct subpopulations (Fig. 5b, Fig. S6), among which the ratio of megakaryocyte/erythroid progenitors (MEPs) was downregulated (Fig. 5c). Next, we determined whether the impairment of MEPs or their upstream lineage, caused this differentiation disorder. To this end, based on the atlas of hematopoietic cells (Fig. S7a–c), subclustering on the hematopoietic progenitors showed three distinct subpopulations, including multipotent progenitors (MPPs), granulocyte/macrophage progenitors (GMPs) & common-myeloid progenitors (CMPs) and MEPs (Fig. 5d, Fig. S7d, e). Remarkably, we found that the ratio of GMPs&CMPs was unchanged and the ratio of MEPs were decreased in *Osx*^*Cre*^*;R26*^*dn/dn*^ mice when compared with its control littermates, and an obvious heterogeneity of MEPs in *Osx*^*Cre*^ and *Osx*^*Cre*^*;R26*^*dn/dn*^ mice was noticed (Fig. 5e). GO analysis showed the impairment of erythrocyte differentiation in the MEPs of *Osx*^*Cre*^*;R26*^*dn/dn*^ mice when compared to their control littermates (Fig. S8a, b). To verify the results from scRNA-seq, we performed flow cytometry analysis on bone marrow. The results showed a significant decrease in LK (cKit^+^Sca^-^) cells, and no difference in LSK or LS^low^K^low^ cells in the bone marrow of *Osx*^*Cre*^*;R26*^*dn/dn*^ mice when compared to control littermates (Fig. 5f, g). Furthermore, in the LK lineage, the CMPs and MEPs were decreased in the BM of the *Osx*^*Cre*^*;R26*^*dn/dn*^ mice (Fig. 5h), which may be related to the anemia and haemorrhagia in osteopetrosis patients.^34^ Additionally, immunohistochemistry staining showed a decrease of SCF^+^ cells, further supporting the impairment of hematopoietic progenitor differentiation (Fig. 5i). Furthermore, we attempted to explore the potential interactions that regulate MEP differentiation in this process and cell communication analysis between osteoblasts and MEPs was performed using the bone marrow scRNA-seq data (Fig. S8c). The results showed that a series of interactions between osteoblasts and MEPs were impaired (Fig. 5j). We combined the impaired osteoblastic ligands and downregulated genes (Fig. 5k), and screened *Pdgfd*, *Vegfb* and another five genes that we inferred to be potential key factors during osteoblast–hematopoietic progenitor crosstalk (Fig. 5l). We then explored whether RAR directly binds to the promoter of the screened genes to regulate their expression, and they were found to contain several potential binding sites, especially *Pdgfd* (Fig. 5m, Fig. S8d), which plays a prominent role in hematopoietic cell growth.^35^ Consistent with this, luciferase assay also indicated RARα showed a significantly enhanced transcriptional function at the *Pdgfd* promoter (Fig. 5n). The typical genes were also verified in the adenovirus-infected *R26*^*dn/dn*^ osteoblasts using RT-qPCR (Fig. 5o, Fig. S8e). Taken together, these results demonstrated that retinoic acid signaling inhibition in osteoblasts may cause osteopetrosis-like erythrocytic and megakaryocytic disorders through impairing the differentiation and function of MEPs via cellular crosstalk.

### Lymphopenia was caused by inhibiting retinoic acid signaling in osteoblasts through impairing B lymphocyte differentiation

**Fig. 6:**
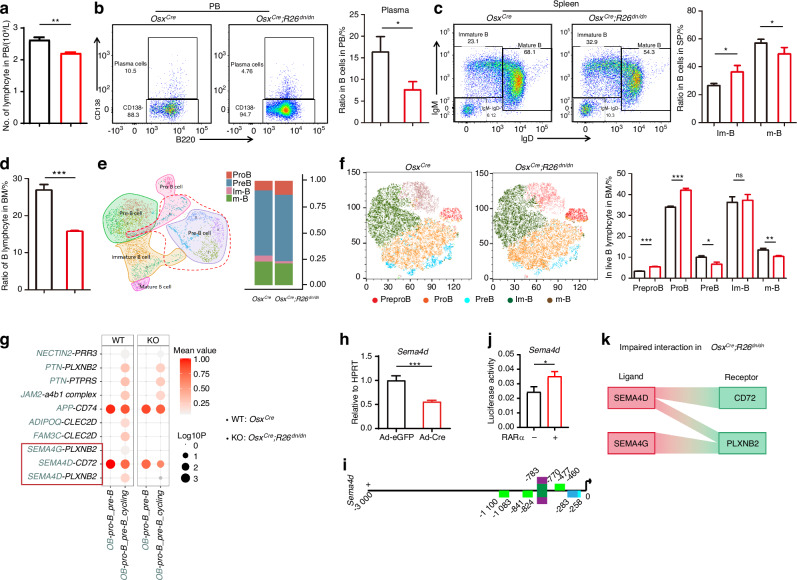
Lymphopenia caused by inhibition of retinoic acid signaling in osteoblasts through impairing B lymphocyte differentiation. **a** Number of lymphocyte in the peripheral blood (PB) of *Osx*^*Cre*^ and *Osx*^*Cre*^*;R26*^*dn/dn*^ mice using hematological analysis. **b** The frequencies of plasma in B lymphocytes in PB from *Osx*^*Cre*^ and *Osx*^*Cre*^*;R26*^*dn/dn*^ mice (*n* = 4 for each group). **c** The frequencies of immature B and mature B cells in B lymphocytes from the spleens of *Osx*^*Cre*^ and *Osx*^*Cre*^*;R26*^*dn/dn*^ mice (*n* = 4 for each group). **d** Ratio of B lymphocytes from the total cells in BM from *Osx*^*Cre*^ and *Osx*^*Cre*^*;R26*^*dn/dn*^ mice (*n* = 4 for each group). **e** Subclustering of the “B lymphocyte” lineage cells from Fig. 6a visualized as a UMAP plot, and the ratio of pro B, pre B, immature B, and mature B cells in the “B lymphocyte” lineage cells. **f** Flow cytometry analysis showing the ratio of prepro B, pro B, pre B, immature B and mature B from the B lymphocytes in BM, visualized with tSNE (*n* = 4 for each group). **g** Cell communication analysis between osteoblasts and pro B and pre B cells in *Osx*^*Cre*^ and *Osx*^*Cre*^*;R26*^*dn/dn*^ mice. **h** The relative mRNA levels of *Sema4d* in the BMSCs of *Osx*^*Cre*^ and *Osx*^*Cre*^*;R26*^*dn/dn*^ mice after culture in osteogenic medium for 7 days. **i** Predicted RAR family binding sites on the screened Semaphorin family (*Sema4d*) human promoters. **j** Luciferase activity analysis for the effect of RARα on the *Sema4d* promoter activity in the 293 T cell line (*n* = 3). **k** The impaired interaction between osteoblasts and B lymphocytes in *Osx*^*Cre*^*;R26*^*dn/dn*^ mice. Error bars are represented as mean±S.D. ns = not significant. *P < 0.05. **P < 0.01. ***P < 0.001. ****P < 0.000 1

Osteopetrosis patients commonly suffer lymphopenia and recurrent infection.^36^ Therefore, we tried to assess the level of lymphocytes in peripheral blood using a hematology analyzer, and a decrease of lymphocyte was exhibited in the *Osx*^*Cre*^*;R26*^*dn/dn*^ mice when compared to their control littermates (Fig. 6a). Then, we further found a decrease of plasma, the important functional cell in B lineages, in peripheral blood using flow cytometry analysis, which may be the cause of frequent infectious complications seen in osteopetrosis (Fig. 6b). Furthermore, the disorders of immature B lymphocytes and mature B lymphocytes in spleen also revealed an impairment in B lymphocyte differentiation (Fig. 6c). Taken together, these results indicated a peripheral impairment of B lymphocyte. Bone marrow is the origin of B lymphocytes, and we further explore whether the disorder was due to the central impairment of B lymphocytic differentiation. Flow cytometry analysis exhibited a significant decrease of B lymphocytes in the bone marrow of *Osx*^*Cre*^*;R26*^*dn/dn*^ mice (Fig. 6d). The result of scRNA-seq also confirmed this (Fig. S9a), mimicking osteopetrosis patients.^37^ We further tried to explore the cause of the decrease of B lymphocytes in bone marrow. Subclustering on the B lymphocytes distinguished pro B lymphocytes, pre B lymphocytes, immature B lymphocytes and mature B lymphocytes (Fig. S9b–d), and a higher ratio of pro B lymphocytes while a lower ratio of mature B lymphocytes were seen in the bone marrow of *Osx*^*Cre*^*;R26*^*dn/dn*^ mice (Fig. 6e). Further flow cytometry analysis showed no significant change in prepro B and pro B lymphocytes, but a decrease in pre B lymphocytes and mature B lymphocytes (Fig. 6f), which indicated that the inhibition of pre B lymphocyte differentiation may be the cause of the total B lymphocyte decrease in bone marrow. Furthermore, KEGG analysis of RNA-seq indicated the impairment of PI3K-Akt signaling, MAPK signaling, cytokine-cytokine receptor interaction and neuroactive ligand receptor interaction. The results further suggested the potential role of cytokines and semaphorin family members (Fig. S9e), which are closely related to PI3K and MAPK pathway activation and reported as neuroactive ligands.^38^ We also further screened and found a decrease in expression of the Semaphorin family in RNA-seq, as well as a decrease in chemokines expression (Fig. S9f). Moreover, we tried to explore the potential interactions that regulate B lymphocyte differentiation in this process and cell communication analysis between osteoblasts and B lymphocytes was performed using the bone marrow scRNA-seq data (Fig. S9g). The results showed that a series of interactions between osteoblasts and B lymphocytes were impaired including SEMA4D/4G-PLXNB2, SEMA4D-CD72 and others (Fig. 6g). Meanwhile, the typical genes were also verified in the adenovirus-infected *R26*^*dn/dn*^ osteoblasts using RT-qPCR (Fig. 6h, Fig. S9h). The ligands were also found to contain several potential binding sites (Fig. 6i, Fig. S9I). Consistent with this, luciferase assay also indicated that RARα showed a significantly enhanced transcriptional function on the *Sema4d* promoter (Fig. 6j). Collectively, these data indicated that inhibition of retinoic acid signaling in osteoblasts could result in osteopetrosis-like impaired B lymphocytes differentiation, which may partially be due to the affected cell-cell crosstalk (Fig. 6k).

In summary, we constructed previously unreported osteopetrosis-like mice with skeletal, hematopoietic, and immune disorders by the inhibition of retinoic acid signaling in osteoblasts, and our data further suggests that retinoic acid signaling in osteoblasts is essential to skeletal, hematopoietic and immune systems during development.

## Discussion

Osteopetrosis was first reported a century ago, and was characterized as “patients with increased bone density”.^2^ Since then, various types of osteopetrosis have been observed, in both children and adults, as having thin, thick, or normal cortical bone, resulting from autosomal dominant or autosomal recessive mutants.^2^ In general, there are some essential phenotypes exhibited in most types of osteopetrosis: skeletal and hematopoietic metabolic disorders, including increased bone density, dwarfism, the brittleness of bone, defects in bone resorption, and hematopoiesis.^39^ In our model, we observed increased imperfectly-formed trabecular bone deposition (increased bone density) with reduced marrow cavity, dwarfism, thin cortical bone with brittle skeleton, impaired osteoclastogenesis, hematopoiesis, and immune dysfunction, which mimics the key phenotypes of osteopetrosis. Thus, we indicated that represents osteopetrosis-like disorder caused by a new way derived from osteoblast impairment.

Retinoic acid signaling is the essential pathway of vitamin A intake, both abnormal activation and inhibition of retinoic acid signaling could lead to craniofacial, skeletal, and hematopoietic disorders.^16^ In the past three decades, some studies have suggested a beneficial effect of vitamin A for bone health, some indicated an association between increased vitamin A intake and osteoporosis, while others have suggested no association or only a weak relationship between increased vitamin A intake and bone fractures. Indeed, many researches have reported an essential role for retinoic acid signaling in development,^40^ while the underlying mechanism of osteoblastic retinoic acid signaling in bone development is far from clear and its role in hematopoietic and immune systems has not been reported. However, there is some evidence supporting a role for retinoid acid signaling as a mediator of osteopetrosis. Patients with the RAR loss of function (LOF) mutant exhibited osteopetrosis-like symptoms.^41,42^ Retinoic acid relieved osteopetrosis symptoms by activating osteoclastogenesis in animal models and some patients.^17,18^ Our previous study indicated that osteoblastic retinoic acid signaling inhibition caused craniofacial deformity.^23^ In this research, we further explored phenotype redouts, such as long bones, and the hematopoietic and immune systems, and increased bone density and brittle bone were detected. Disorders in hematopoietic progenitors and B lymphocytes differentiation were shown, which can lead to anemia, haemorrhagia and frequent infectious complications. These results indicated that osteoblastic retinoic acid signaling inhibition could lead to osteopetrosis-like metabolic disorders, and highlights the importance of osteoblastic retinoic acid signaling in development and sheds a new light on potential osteopetrosis therapy.

Skeletal disorders can lead to the shrinkage of the marrow cavity, and hematopoietic disorder could impair osteoclastogenesis. Thus, it is very difficult to say which disorder is the etiology, but undoubtedly, there could exist a vicious circle between increased poorly remodeled trabecular bone and impaired hematopoiesis, which may cause a greater narrowed cavity during development until death. To this end, many previous studies highlighted and focused on osteoclastogenesis.^43^ A series of murine models targeted at osteoclastic function were constructed to mimic osteopetrosis,^44^ the edited genes of which can divided into two main groups: proteins expressed in osteoclasts (e.g. CLCN7) and proteins that induce osteoclastogenesis (e.g. M-CSF). CLCN7 encodes the voltage-gated chloride channel 7(CLC-7), and this chloride gated channel is specific to osteoclasts, and is essential for osteoclastic activation through the regulation of cytoplasmic pH.^12^ M-CSF is an essential secretory protein produced and secreted by other bone marrow cells in the microenvironment, which is necessary for osteoclastic differentiation.^45^ In summary, currently, most murine models of osteopetrosis are the result of defects in osteoclastic differentiation, but actually comparable defects were not always documented in human osteopetrosis.^2^ Our results found that osteoblastic disorders impaired osteogenesis, osteoclastogenesis, hematopoiesis and immune system, and eventually exhibited an osteopetrosis-like phenotype. We compared our model with other established models for osteoporosis (Table S3) and found that they were similar to some degree.^2^ Thus, we propose that osteoblastic disorders may be one of the causes of osteopetrosis. Indeed, known as the “osteoclast non-autonomous form”,^2^ previous research on osteopetrosis has proposed a potential role for the osteoblast lineage, which is the key lineage involved in bone marrow microenvironment formation and maintenance.^2^ However, the underlying mechanism remained unclear. Recently, many researchers have reported that during bone development, osteoblasts can regulate osteoclastogenesis through cell-cell crosstalk,^46^ but whether it happens in osteopetrosis progression is unknown. A recent study reported that the inactivation of *Rankl* specifically in osteoblast lineage cells from mice with the use of an Osterix-Cre transgene, results in typical osteopetrosis in the tibia, which indicated a potential role for this pathway.^47^ Taken together, in our study, we believe that the *Osx*^*Cre*^*;R26*^*dn/dn*^ mice exhibit osteopetrosis-like disorder in a previously unreported osteoblast-originated “osteoclast non-autonomous form”, which mimics osteopetrosis functionally rather than genetically. It is also expected to be verified from more aspects and become an alternative research model for osteoporosis in the future.

Focusing on the thin cortical bone and increased trabecular bone, which are common phenotype in osteopetrosis, here we take a classic osteopetrosis mouse model, *Csf1*^−^^/−^ mice, as an example. In *Csf1*^*−/−*^ mice, researchers also noticed thin cortical bone and increased trabecular bone, the decrease of osteoclastogenesis resulted in the increased trabecular bone, and Xie H et al. reported that the decreased pre-Oc secreted PDGFB inhibited osteogenesis in the cortical bone and thus resulted in thin cortical bone.^26^ Therefore, we also tested PDGFB expression in the cortical bone of our mouse model and we found that it was also decreased in the *Osx*^*Cre*^*;R26*^*dn/dn*^ mice compared to the control littermates. Taken together, we inferred that in the trabecular bone of *Osx*^*Cre*^*;R26*^*dn/dn*^ mice, osteoblastic retinoic acid signaling inhibition impaired osteoclastogenesis through osteoblast-osteoclast crosstalk that caused the increased imperfectly-formed trabecular bone; while in the cortical bone of *Osx*^*Cre*^*;R26*^*dn/dn*^ mice, the inhibited osteoclast function caused by osteoblast dysfunction might also decrease the expression of PDGFB and thus further inhibit osteogenesis in the cortical bone. This positive feedback effect on osteogenesis downregulation and the cell-autonomous effect we reported in Fig. 3 might together result in thin cortical bone.

In this study, we found SFRP4 could partially rescue the decreased osteogenic activity caused by osteoblastic retinoic acid signaling inhibition in vitro. SFRP4 is an antagonist of Wnt signaling, knockout of *Sfrp4* in osteoblast resulted in reduced bone formation, and mice deficient in *Sfrp4* exhibited thin cortical bone but increased bone density.^48^ As wnt proteins facilitate osteogenic activity through suppressing apoptosis in osteoblast precursor cells prior to determination of cell differentiation, we inferred that there might be a stem cell depletion at an early stage in the development of *Osx*^*Cre*^*;R26*^*dn/dn*^ mice that results in imperfectly-formed trabecular bone deposition, which is consistent with the phenotype in a previous study in which impaired clonal multipotency was found in osteoblast progenitors.^31^ Indeed, expression of some other osteogenic genes were also decreased in the osteoblasts from our mutant mice, we highlight *Sfrp4* as a typical gene that might lead to the osteopetrosis-like phenotype. Taken together, we suggest that osteoblastic retinoic signaling inhibition could affect bone anabolism in a cell-autonomous manner, which may be related to the reduction of *Sfrp4* expression.

In our results, the *Prx1*^*Cre*^*;R26*^*dn/dn*^ mice exhibited decreased bone density, while *Osx*^*Cre*^*;R26*^*dn/dn*^ mice exhibited osteopetrosis characterized by thin cortical bone but increased bone density. The different phenotype of *Prx1*^*Cre*^*;R26*^*dn/dn*^ and *Osx*^*Cre*^*;R26*^*dn/dn*^ mice drew our attention to the different function of the two cell lineages. At the level of organ development, Prx1 Cre targets the limbs and not the appendicular skeleton,^49^ which could be also responsible for the phenotype. At the level of cellular function, lineage tracing found the different location of the two cell lineages, which was consistent with a previously reported scRNA-seq data.^29^ Further focusing on bone turnover, as for the osteogenesis, immunofluorescence showed a decrease of OSX^+^ cells in the femur of *Prx1*^*Cre*^*;R26*^*dn/dn*^ mice compared to their control littermates, indicating the inhibition of osteogenesis (Fig. S10a). SFRP4 staining also indicated the impairment of bone anabolism in the cortical bone of *Prx1*^*Cre*^*;R26*^*dn/dn*^ mice compared to their control littermates (Fig. S10b). As for osteoclastogenesis and hematopoiesis, we focused on the ability of cell-cell crosstalk. We retrieved two previously reported scRNA-seq data sets,^29^ which are CD45^-^Ter119^-^CD31^-^Ai9^+^ cells from the femur of *Prx1*^*Cre*^*;R26*^*ai9*^ (Prx-Cre_lineage, 725 cells) and *Osx*^*Cre*^*;R26*^*ai9*^ (Osx-Cre_lineage, 909 cells) mice (including bone marrow and bone fragments), for analysis (Fig. S10c). Four main clusters were mapped to BMSCs (expressing *Scf* and *Cxcl12*), osteoblasts (Ob, expressing *Bglap* and *Col1a1*), periosteal mesenchymal stromal cells (PMSCs, expressing *Dcn* and *Col3a1*) and chondrocyte-like cells (chondrocytes, expressing *Sox9* and *Acan*) according to a previous study (Fig. S10d). Initially, differential genes were screened and some secretory proteins, such as MMP13 which is important in osteoblast-osteoclast crosstalk,^50^ were found higher in the Osx-Cre_lineage when compared with the Prx-Cre_lineage (Fig. S10e), thus we inferring that Osx-Cre_lineage cells might have a stronger impact on the neighbouring cells in the bone microenvironment through cell-cell crosstalk. Thus, gene ontology (GO) analysis was performed focusing on these highly-expressed genes of the Osx-Cre_lineage (Fig. S10f, g). As a result, Osx-Cre_lineage showed advantage on cell-nonautonomous crosstalks, including macrophage/osteoclast activation (such as complement activation), angiogenesis and immune system process, when compared with Prx-Cre_lineage (Fig. S10f). Focusing on BMSCs&Obs clusters, Osx-Cre group showed upregulated cell-cell crosstalk activity compared to Prx-Cre group (Fig. S10g). We further performed cellphone analysis between Osx-Cre/Prx-Cre lineage cells and pre-osteoclasts & osteoclasts in WT or KO sets using the scRNA-seq data mentioned above Fig. S10H). Many pairs were affected in the KO group, among which C3-C3AR1 was impaired in the BMSCs in Osx-Cre_KO group but not in the BMSCs in Prx-Cre KO group (Fig S10i). Taken together, we inferred that the diffenrent phenotype between *Osx*^*Cre*^*;R26*^*dn/dn*^ and *Prx1*^*Cre*^*;R26*^*dn/dn*^ mice in trabecular bone might due to different abilities of crosstalk of the two lineages. Therefore, we tried to verify the cell-cell crosstalk function histologically. For example, although the osteoclasts in the *Prx1*^*Cre*^*;R26*^*dn/dn*^ mice were also a little bit fewer compared with their control littermates, the impairment of bone resorption seemed not as sereve as that in the *Osx*^*Cre*^*;R26*^*dn/dn*^ mice (Fig. S10j). Moreover, the change of PDGFB expression in the *Prx1*^*Cre*^*;R26*^*dn/dn*^ mice compared to their control littermates was also much slighter than the change in the *Osx*^*Cre*^*;R26*^*dn/dn*^ mice compared to their control littermates (Fig. S10k). Furthermore, the number of SCF^+^ cells didn’t showed significant change in the *Prx1*^*Cre*^*;R26*^*dn/dn*^ mice (Fig. S10l), thus we further inferred that some crosstalks might be impaired in the *Osx*^*Cre*^*;R26*^*dn/dn*^ mice, but may not in the *Prx1*^*Cre*^*;R26*^*dn/dn*^ mice. Meanwhile, we also inferred that *Osx*^*Cre*^*;R26*^*dn/dn*^ mice might suffer a severe impairment of osteoblast-dominant cell crosstalk than *Prx1*^*Cre*^*;R26*^*dn/dn*^ mice, which might lead to the diffenrent phenotype in trabecular bone. Although which cell cluster results in it and whether it could be marked, still need further exploration. Additionally, multiple cell types including stromal cells, adipocytes and perivascular cells could also be targeted by Osx Cre,^51^ thus in order to further validate that inhibition of retinoic acid signaling in osteoblasts is responsible for the osteopetrotic phenotype, Ocn-Cre or Col1a1-Cre is useful for further exploration.

Recently, osteoimmunology has received considerable attention.^52^ In the past, most researches have focused on the effect of the immune system on bone metabolism. Recently however, we have also witnessed the discovery of the effects of bone cells on immune regulation, including the function of osteoprogenitor cells in hematopoietic stem cell regulation.^52^ Osteogenic factors, such as IL-19 secreted by osteocytes^53^ and IL-34 secreted by osteoclasts,^54^ can regulate immune system development. However, studies on the immunological changes regulated by osteoblasts were very rare and its underlying mechanism was far from clear. Our model specifically inhibited retinoic acid signaling in osteoblasts and found impaired of hematopoietic progenitor and B lymphocyte differentiation via cell crosstalk, and whose phenotypes were osteopetrosis-like, indicating that it may be a suitable therapeutic model for osteoimmunology study. Moreover, we highlight osteoblastic retinoic acid signaling in hematopoietic progenitor and B lymphocyte differentiation, which is represents a novel finding, but the detailed mechanism of osteoblast-dominant cell-cell crosstalk needs further exploration. Taken together, we demonstrated that osteoblastic retinoic acid signaling may regulate osteoclastogenesis and hematopoiesis through cell-cell crosstalk in the bone marrow microenvironment during development and that aberrant osteoblastic retinoic acid signaling may result in osteopetrosis.

In summary, we unveiled previously unreported pathogenesis of osteopetrosis-like disorder induced by osteoblastic retinoic acid signaling inhibition, which emphasizes the role of retinoic acid in osteoblasts during skeletal, hematopoietic and immune system development, and sheds new insights into osteopetrosis.

## Methods

Some methods are described in the Supplementary.

### Statistical analyses

Statistical analyses were performed using GraphPad Prism 6.01 software (GraphPad Software Inc., La Jolla, CA, USA). Cell-based experiments were performed at least twice. Animals were randomized into different groups and at least three mice were used for each group, unless otherwise stated. The Kolmogorov-Smirnov (K-S) test was used to test the correlation between the control set and experimental set. All quantitative data are presented as the mean±S.D. Student’s t-test was used for statistical evaluations of two group comparisons. *P* < 0.05 was considered statistically significant.

## Supplementary information

Appendix

## Data Availability

All data and genetic material used for this article are available upon request to the corresponding authors. Source data are provided with this article.
