# Supplementary material for: Osteopetrosis-like disorders induced by osteoblast-specific retinoic acid signaling inhibition in mice
Source: Bone Res. 2024 Oct 17;12:61. doi: 10.1038/s41413-024-00353-5 (PMC11487257; doi:10.1038/s41413-024-00353-5)
Supplement: Supplementary file 1 — Appendix [file 41413_2024_353_MOESM1_ESM.docx]

**A new osteopetrosis-like mice model induced by inhibiting retinoic acid signaling in osteoblasts**

Siyuan Sun^#1^, Yuanqi Liu^#1^, Jiping Sun^#3^, Bingxin Zan^2^, Yiwen Cui^1^, Anting Jin^1^, Hongyuan Xu^1^, Xiangru Huang^1^, Yanfei Zhu^1^, Yiling Yang^1^, Xin Gao^1^, Tingwei Lu^1^, Xinyu Wang^1^, Jingyi Liu^1^, Lei Shen^3^, Qinggang Dai*^2^, Lingyong Jiang*^1^

**Supplementary Figures**


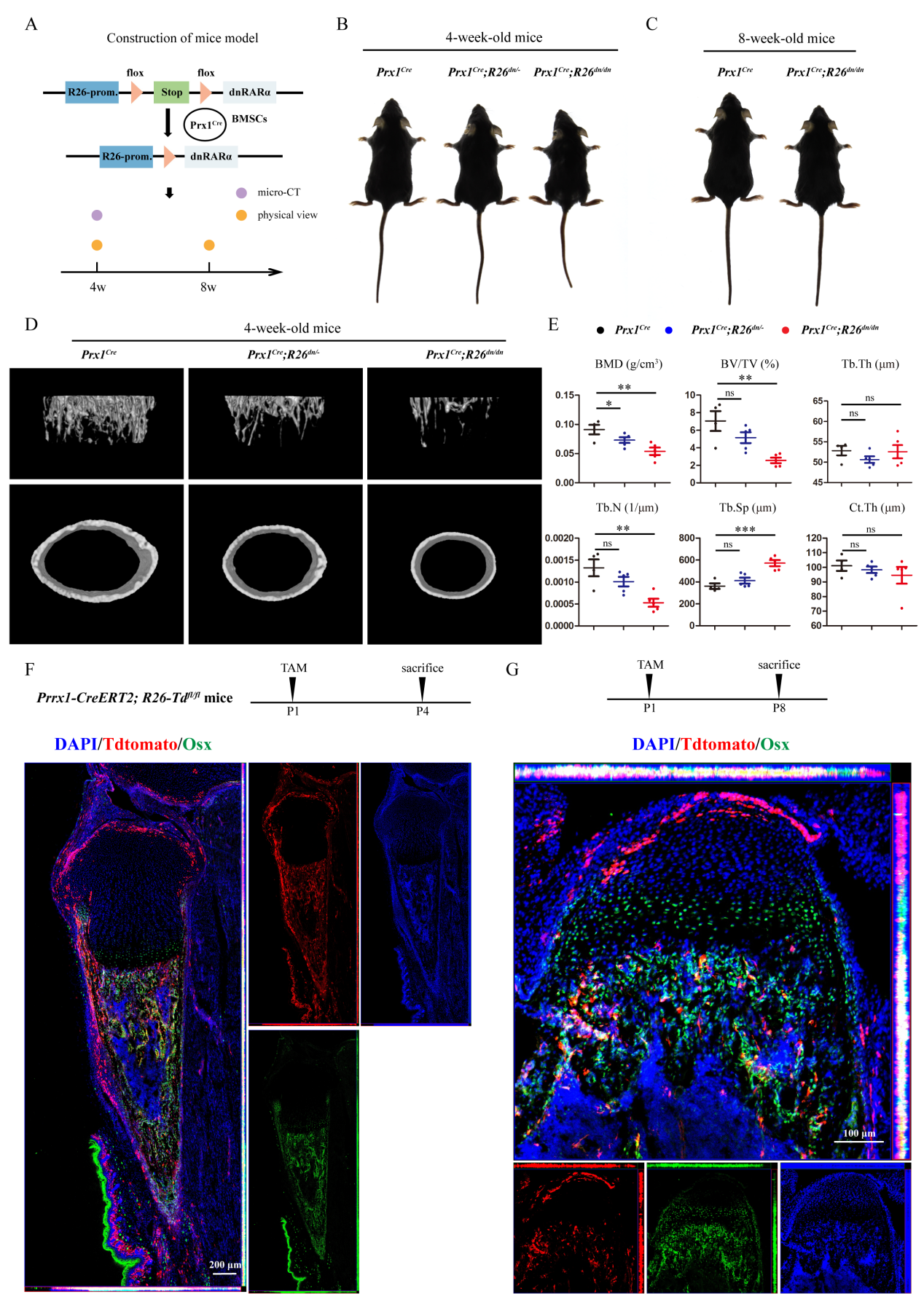


**Supplementary Figure 1. Retinoic signaling inhibition in BMSCs did not result in osteopetrosis-like skeletal disorders.** (A) Illustration of mice model construction and phenotype analysis. (B) Representative views of 4-week-old *Prx1^Cre^*, *Prx1^Cre^;R26^dn/-^* and *Prx1^Cre^;R26^dn/dn^* mice. (C) Representative views of 8-week-old *Prx1^Cre^* and *Prx1^Cre^;R26^dn/dn^* mice. (D) Micro-CT images of trabecular bone and cortical bone of femurs from 4-week-old *Prx1^Cre^*, *Prx1^Cre^;R26^dn/-^* and *Prx1^Cre^;R26^dn/dn^* mice. (E) Quantitative parameters of micro-CT of distal femurs from 4-week-old mice, including bone volume per tissue volume (BV/TV), trabecular thickness (Tb.Th.), trabecular number (Tb.N.), trabecular space (Tb.Sp.), and cortical thickness (Ct.Th). (F-G) The different location of Prrx1+ lineage and Osx+ cells using Prrx1 lineage tracing and Osterix immunofluorescence. Tamoxifen was induced on P1 and the mice was sacrificed on P4 in (F) and tamoxifen was induced on P1 and the mice was sacrificed on P8 in (G). Error bars are represented as mean±SD. ns = not significant. *P < 0.05. **P < 0.01. ***P < 0.001.


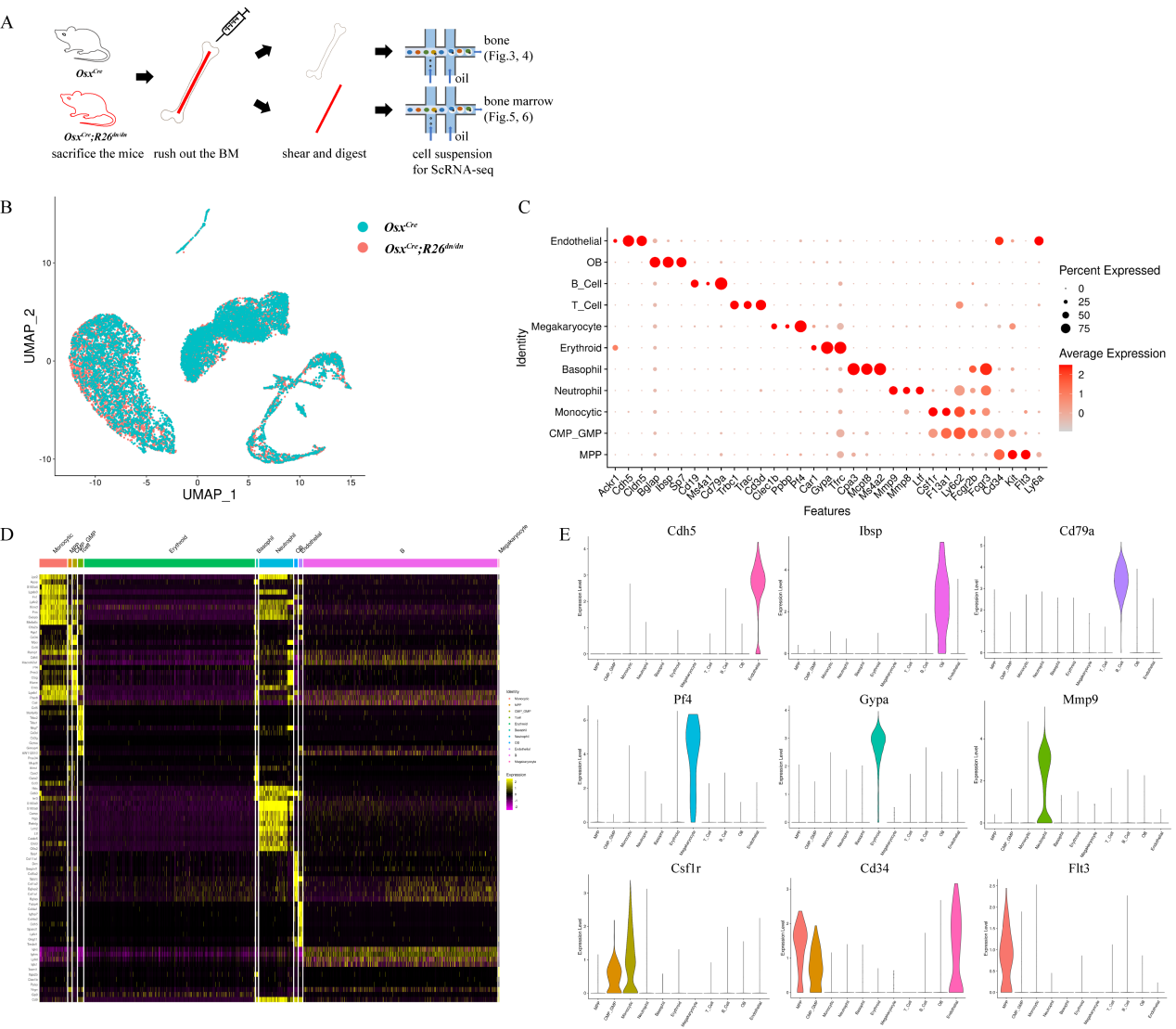


**Supplementary Figure 2. A single cell atlas of the bone of *Osx^Cre^* mice and *Osx^Cre^;R26^dn/dn^* mice.** (A) Flowchart showing the preparation of single-cell RNA sequencing (scRNA-seq) samples from mouse femurs without bone marrow and samples of mouse bone marrow. (B) A total of 11060 cells from *Osx^Cre^* mice (green) and a total of 10552 cells from *Osx^Cre^*;*R26^dn/dn^* mice (red) identified by scRNA-seq were visualized with UMAP. (C) Bubble map showing differentially-expressed genes for each cluster. (D) Heat map showing the scaled expression of differentially-expressed genes for each cluster. (E) Feature plots of the expression levels of marker genes of endothelial cells, osteolineage cells, B lymphocytes, Megakaryocytes, Erythroid cells, Neutrophils, Monocytic cells, CMP & GMPs and MPPs, representatively.


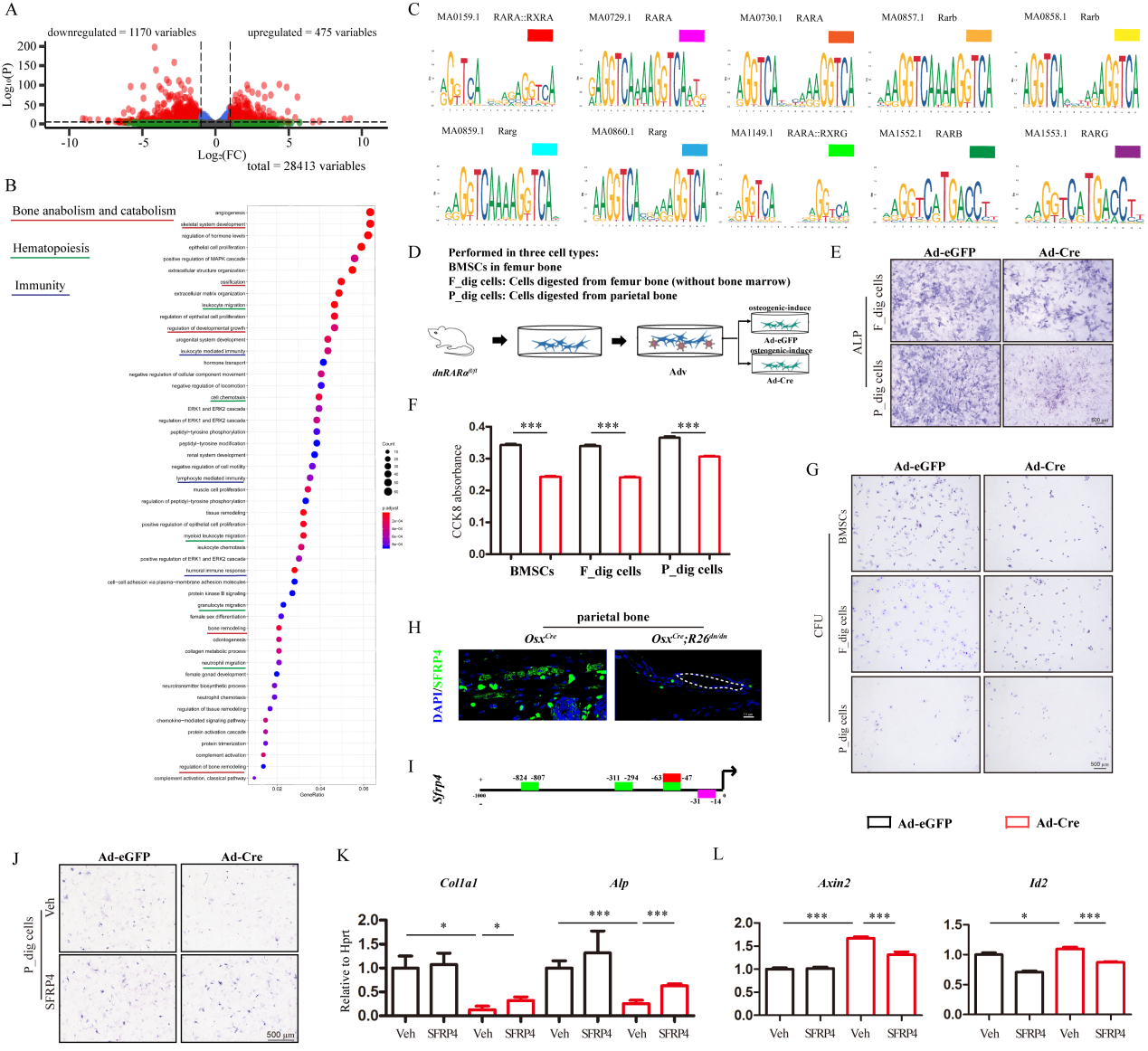


**Supplementary Figure 3.** (A) RNA-seq analysis of BMSCs of *Osx^Cre^* and *Osx^Cre^;R26^dn/dn^* mice. Volcano plot displays global gene expression in the *Osx^Cre^* and *Osx^Cre^*;*R26^dn/dn^* sets. Two-sided test. Left represents downregulated genes, right represents upregulated genes. (B) Gene ontology (GO) enrichment analysis of downregulated genes (>2 fold) in the *Osx^Cre^*;*R26^dn/dn^* set. Red lines highlight the processes related to bone anabolism and catabolism. Green lines highlight the processes related to hematopoiesis. Blue lines highlight the processes related to immunity. (C) Predicted motifs of RARE. (D) Flowchart showing that three cell types, including BMSCs, cells digested from femur bone without bone marrow (F_dig cells) and cells digested from parietal bone (P_dig cells). (E) ALP staining of *Osx^Cre^* and *Osx^Cre^;R26^dn/dn^* mouse F_dig cells and P_dig cells after culture in osteogenic medium for 7 days. (F) Cell proliferation analysis of *Osx^Cre^* and *Osx^Cre^;R26^dn/dn^* mouse BMSCs, F_dig cells and P_dig cells after culture for 7 days by CCK8 assay. (G) Colony formation assay of Ad-eGFP or Ad-Cre infected *R26^dn/dn^* mouse BMSCs, F_dig cells and P_dig cells after culture for 7 days. (H) SFRP4 expression in the parietal bone of *Osx^Cre^* and *Osx^Cre^;R26^dn/dn^* mouse by IF staining. (I) Predicted RAR family binding sites on the screened osteoblastic gene *Sfrp4* promoters. (J) ALP staning of the Ad-eGFP or Ad-Cre infected and 0.1 μg/mL SFRP4 recombinant protein rescued P_dig cells from *R26^dn/dn^* mouse after culture in osteogenic medium for 7 days. (K) The mRNA expression of *Col1a1* and *Alp* in the Ad-eGFP or Ad-Cre infected and 0.1 μg/mL SFRP4 recombinant protein rescued P_dig cells from *R26^dn/dn^* mouse after culture in osteogenic medium for 7 days by RT-qPCR. (L) The mRNA expression of *Axin2* and *Id2* in the Ad-eGFP or Ad-Cre infected and 0.1 μg/mL SFRP4 recombinant protein rescued BMSCs from *R26^dn/dn^* mouse after culture for 7 days by RT-qPCR. Error bars are represented as mean±SD. *P < 0.05. **P < 0.01. ***P < 0.001.


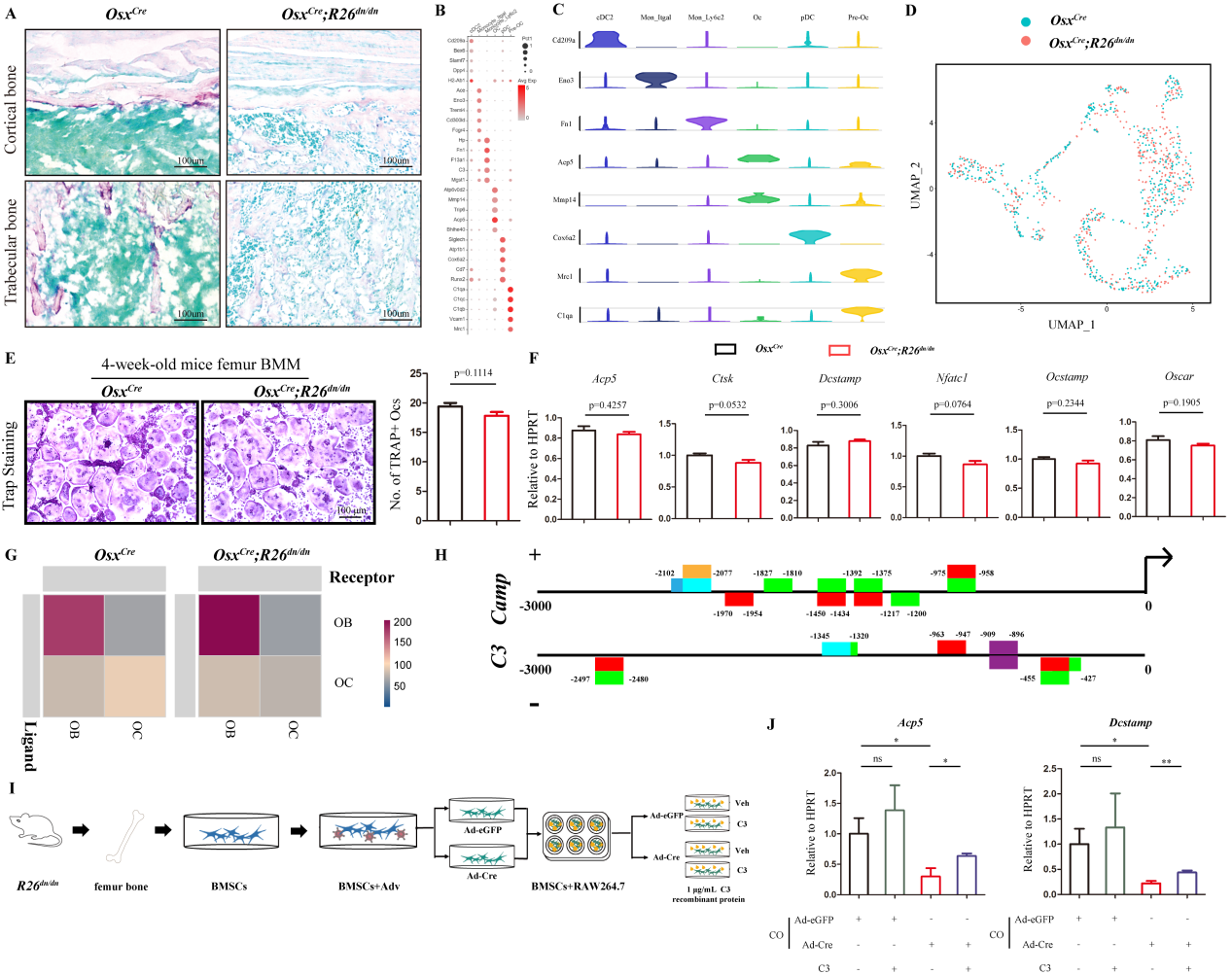


**Supplementary Figure 4.** (A) TRAP staining of femur cortical bone (upper panel) and trabecular bone (lower panel) from 7-day-old *Osx^Cre^;R26^dn/dn^* mice and their control littermates. (B) Subclusters of the monocytic cells. Bubble map showing differentially-expressed genes for each subcluster. (C) Feature plots of the expression levels of marker genes of conventional dendritic cells (cDC), Monocytes, Osteoclasts (Oc), plasmacytoid dendritic cells (pDC) and pre-osteoclasts (pre-Oc). (D) Monocytic cells of *Osx^Cre^* (green) and *Osx^Cre^;R26^dn/dn^* (red) mice were visualized with UMAP, respectively. (E) TRAP staining of BMMs from 4-week-old *Osx^Cre^* and *Osx^Cre^;R26^dn/dn^* mice after inducing with M-CSF for 7 days and inducing with M-CSF and RANKL for 5 days. (F) The relative mRNA levels of osteoclast-specific genes *Acp5, Ctsk,* *Dcstamp, Nfatc1, Ocstamp* and *Oscar* in BMMs from 4-week-old *Osx^Cre^* and *Osx^Cre^;R26^dn/dn^* mice after inducing with M-CSF for 7 days and inducing with M-CSF and RANKL for 5 days (n=9). (G) Cell communication analysis between osteoblasts and osteoclasts & pre-osteoclasts in *Osx^Cre^* and *Osx^Cre^;R26^dn/dn^* mice. (H) Predicted RAR family binding sites on the screened osteoblastic ligands *Camp* and *C3* human promoters, representatively. (I) Coculture of Ad-eGFP or Ad-Cre infected *dnRARα^fl/fl^* preosteoblasts and RAW264.7, intervened with Complement C3. (J) The relative mRNA levels of osteoclast-specific genes *Acp5* and *Dcstamp* of the cocultured RAW264.7. Error bars are represented as mean±SD. ns = not significant. *P < 0.05. **P < 0.01.


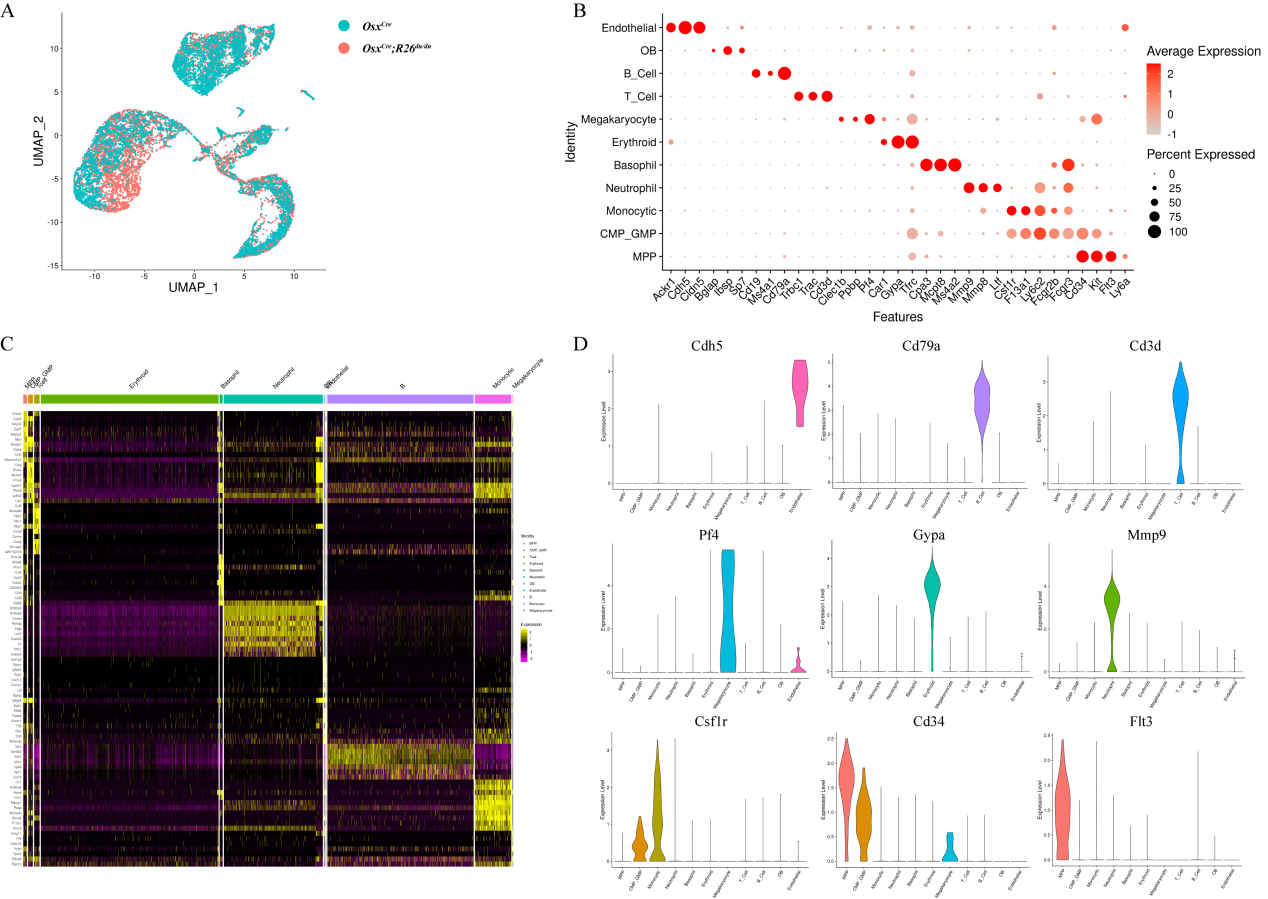


**Supplementary Figure 5. A single cell atlas of the bone marrow of *Osx^Cre^* mice and *Osx^Cre^;R26^dn/dn^* mice.** (A) A total of 6827 cells from *Osx^Cre^* mice (green) and a total of 9151 cells from *Osx^Cre^*; *R26^dn/dn^* mice (red) identified by scRNA-seq were visualized with UMAP. (B) Bubble map showing differentially-expressed genes for each cluster. (C) Heat map showing the scaled expression of differentially-expressed genes for each cluster. (D) Feature plots of the expression levels of marker genes of endothelial cells, B lymphocytes, T lymphocytes Megakaryocytes, Erythroid cells, Neutrophils, Monocytic cells, CMP & GMPs and MPPs, representatively.


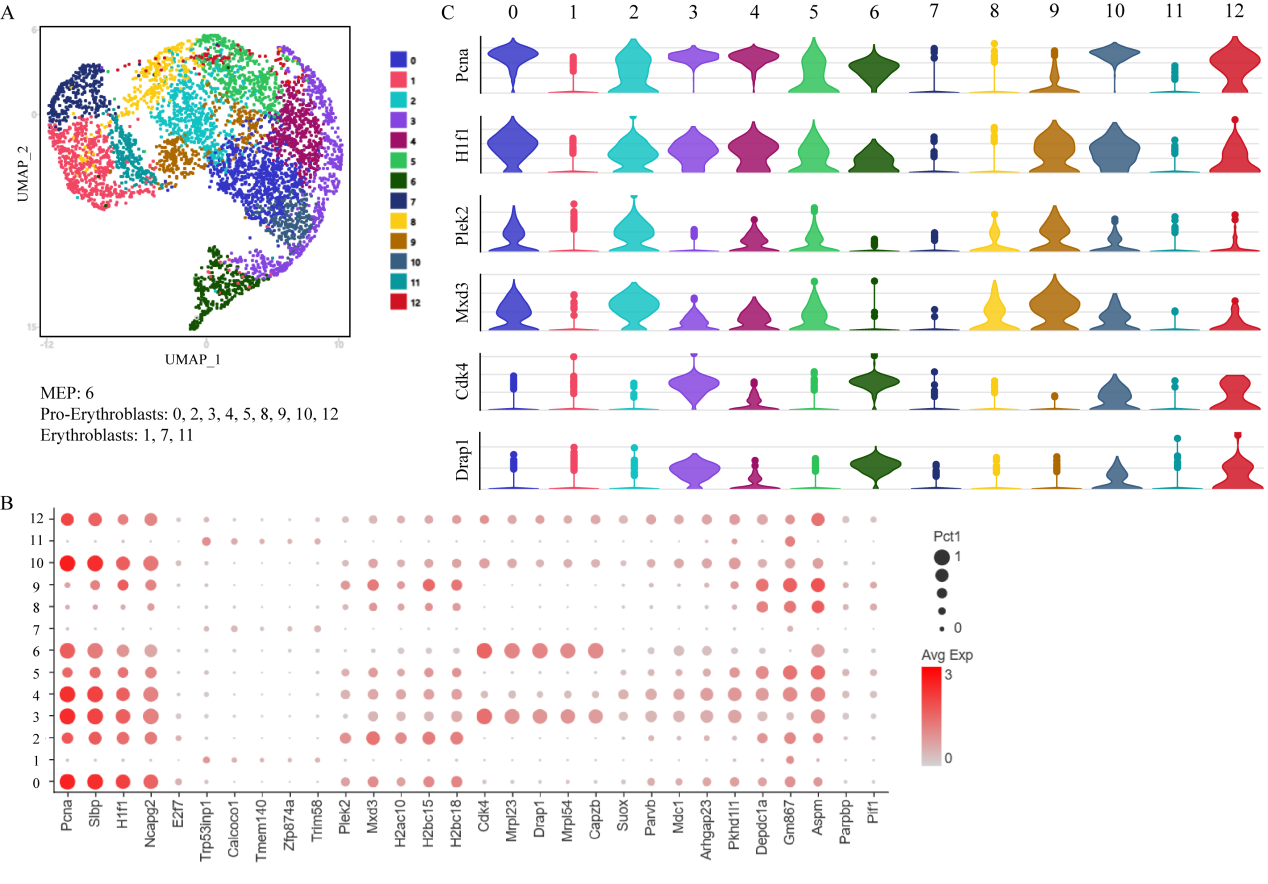


**Supplementary Figure 6. Subclusters of the erythroid cells.** (A) Atlas of erythroid cells. Twelve cell populations were defined and distinguished by color. Each point represents an individual cell. (B) Bubble map showing differentially-expressed genes for each subcluster. (C) Feature plots of the expression levels of marker genes of MEP, Pro-Erythroblasts and Erythroblasts.


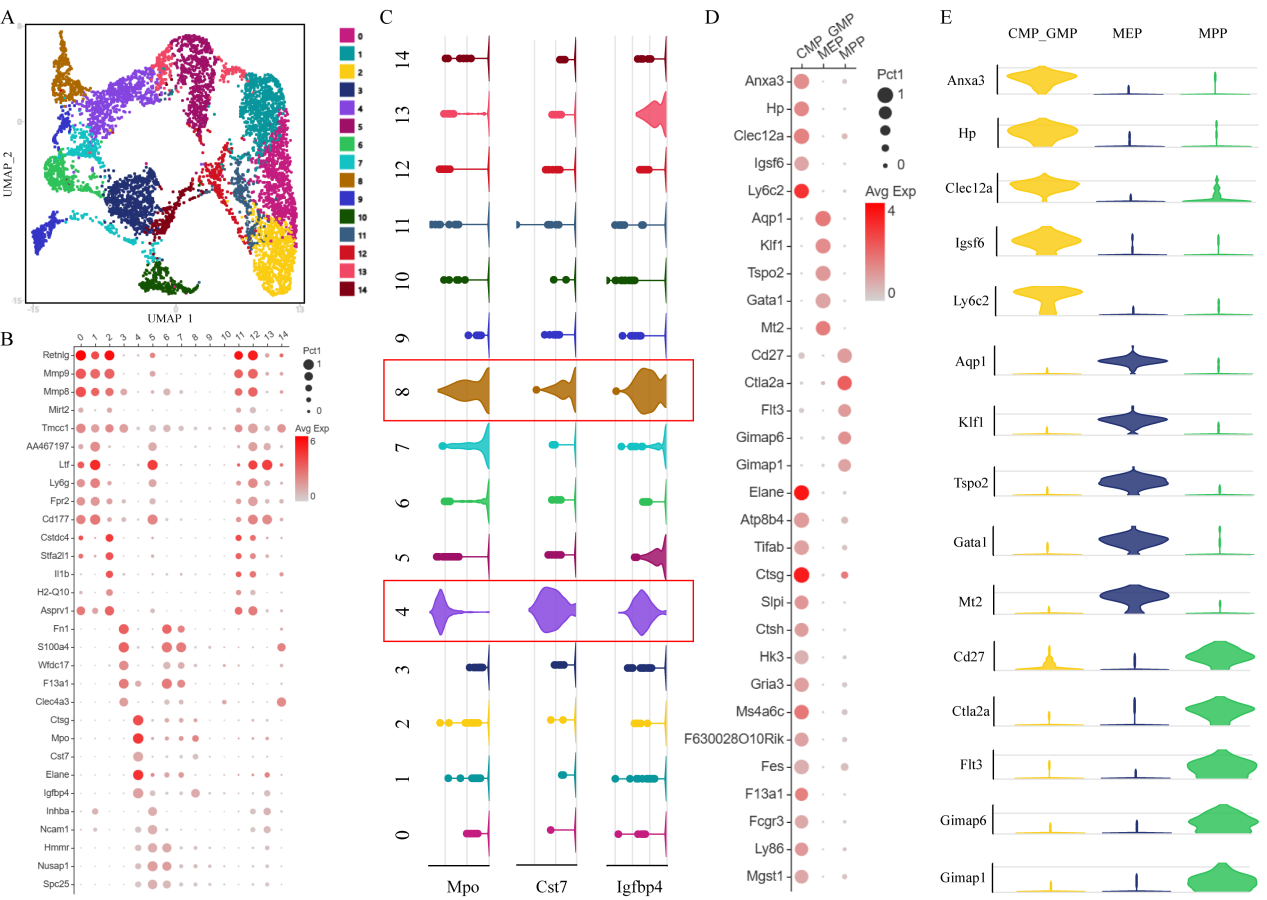


**Supplementary Figure 7. Subclusters of the hematopoietic cells and hematopoietic progenitors.** (A) Atlas of hematopoietic cells. Fourteen cell populations were defined and distinguished by color. Each point represents an individual cell. (B) Bubble map showing differentially-expressed genes for each subcluster. (C) Feature plots of the expression levels of marker genes of hematopoietic progenitors. Red frames indicate subclusters 4 and 8 are hematopoietic progenitors. (D) Bubble map showing differentially-expressed genes for each subcluster in the atlas of hematopoietic progenitors. (E) Feature plots of the expression levels of marker genes of CMP&GMP, MEP and MPP.


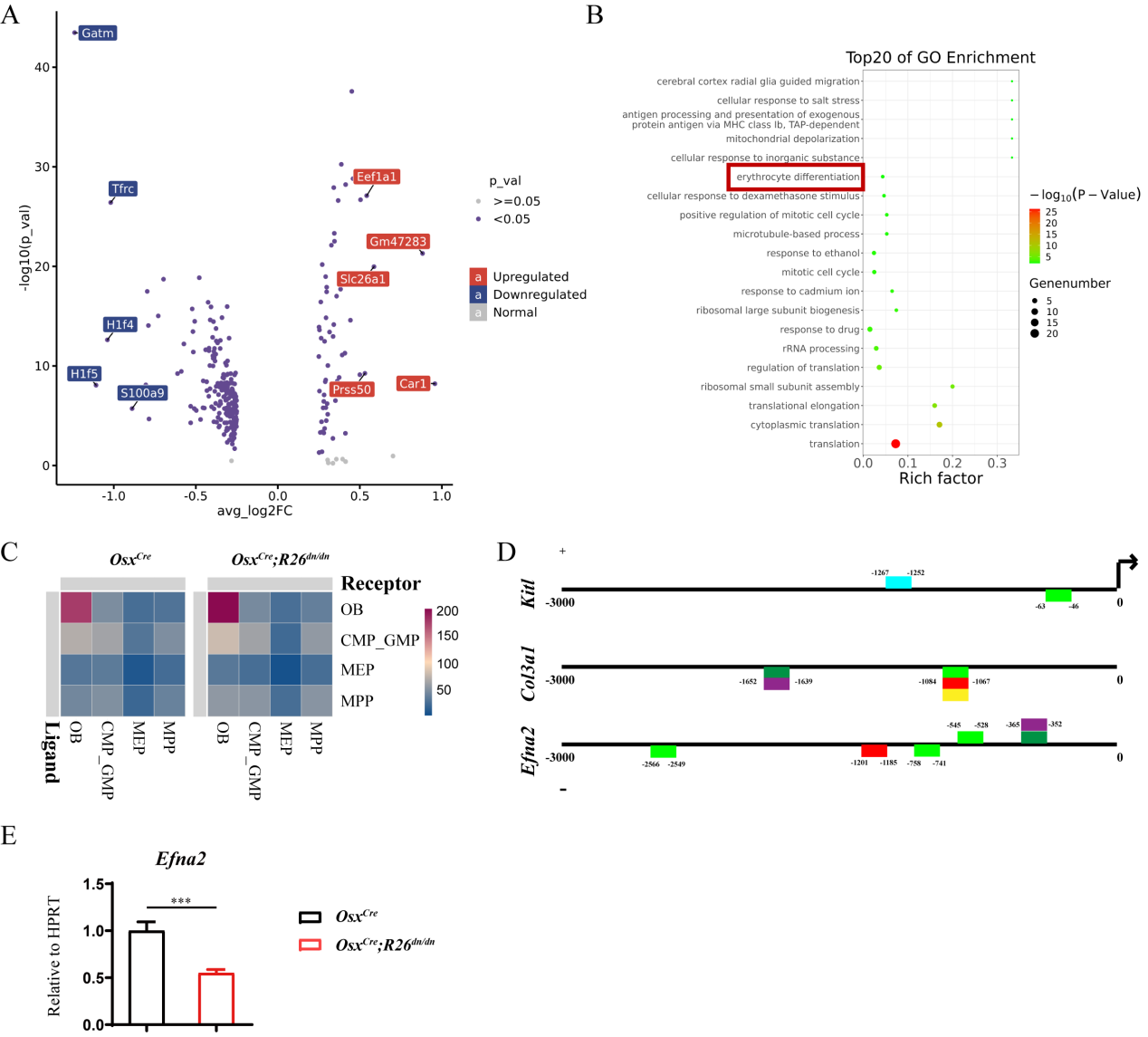


**Supplementary Figure 8.** (A) Volcano plot displays global gene expression in the *Osx^Cre^* and *Osx^Cre^; R26^dn/dn^* MEPs using ScRNAseq data. (B) The GO terms are visualized in a bubble plot. (C) Cell communication analysis between osteoblasts and hematopoietic progenitors in *Osx^Cre^* and *Osx^Cre^;R26^dn/dn^* mice. (D) Predicted RAR family binding sites on the screened osteoblastic ligands *Kitl*, *Col3a1* and *Efna2* human promoters, representatively. (E) The relative mRNA levels of osteoblastic ligand *Efna2* in BMSCs of *Osx^Cre^* and *Osx^Cre^;R26^dn/dn^* mice after culture in osteogenic medium for 7 days. Error bars are represented as mean±S.D. ***P < 0.001.


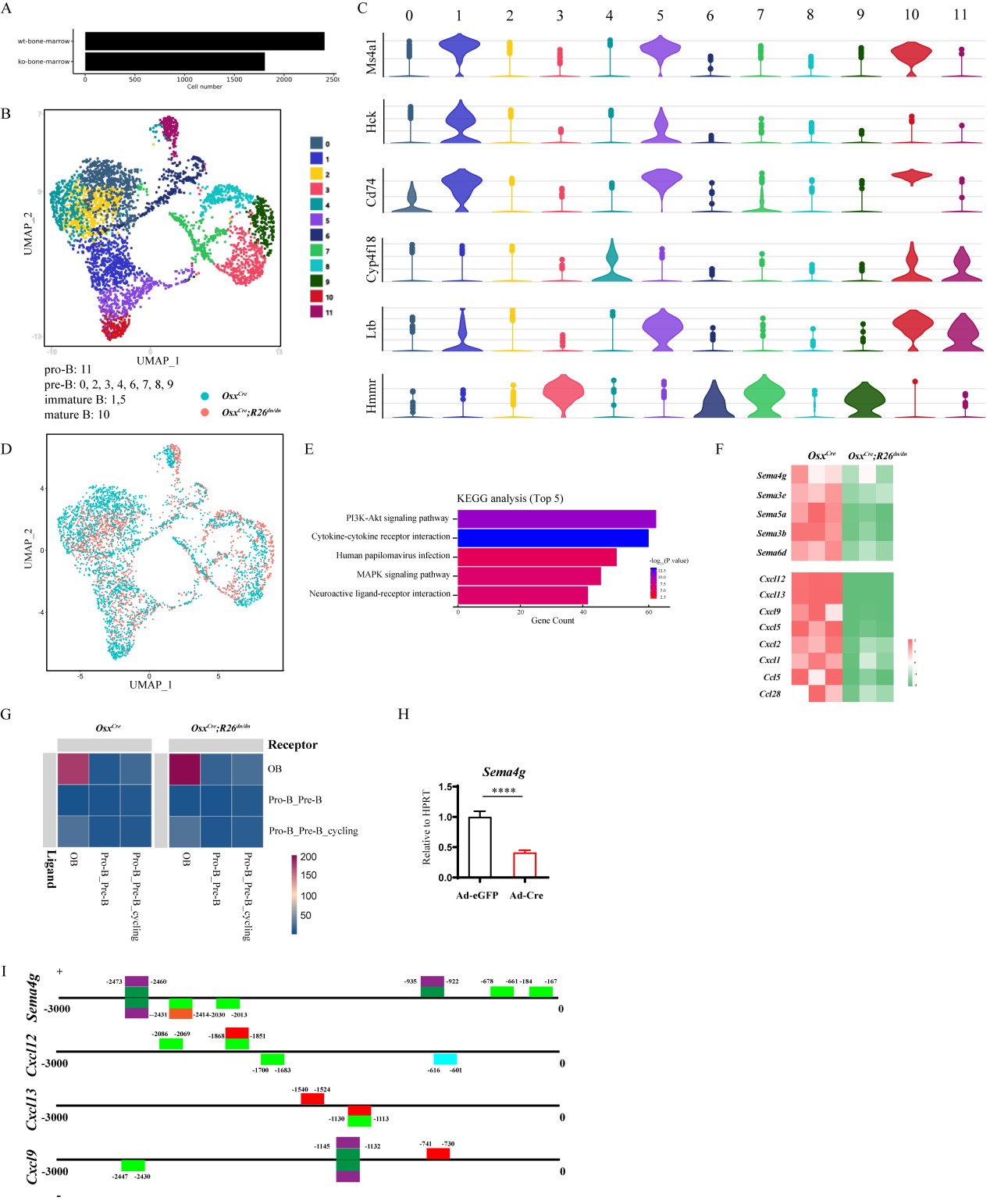


**Supplementary Figure 9. Subclusters of the B lymphocytes and cellphone between osteoblasts and B lymphocytes in *Osx^Cre^* and *Osx^Cre^;R26^dn/dn^* mice.** (A) Number of B lymphocytes in the bone marrow of *Osx^Cre^* and *Osx^Cre^;R26^dn/dn^* mice. (B) Atlas of B lymphocytes. Fourteen cell populations were defined and distinguished by color. Each point represents an individual cell. (C) Feature plots of the expression levels of marker genes of pro-B, pre-B, immature B and mature B lymphocytes. (D) B lymphocytes of *Osx^Cre^* (green) and *Osx^Cre^;R26^dn/dn^* (red) mice were visualized with UMAP, respectively. (E) KEGG analysis of the downregulated genes (>2 fold) in the *Osx^Cre^;R26^dn/dn^* osteoblasts RNA-seq set compared to *Osx^Cre^* set (Top 5). (F) Heatmap analysis of the Semaphorin gene family and chemokines in the *Osx^Cre^* and *Osx^Cre^;R26^dn/dn^* osteoblasts RNA-seq sets. (G) Cell communication analysis between osteoblasts and B lymphocytes in *Osx^Cre^* and *Osx^Cre^;R26^dn/dn^* mice. (H) The relative mRNA levels of *Sema4g* in the BMSCs of *Osx^Cre^* and *Osx^Cre^;R26^dn/dn^* mice after culture in osteogenic medium for 7 days. (I) Predicted RAR family binding sites on *Sema4g* and the chemokines (*Cxcl12*, *Cxcl13* and *Cxcl9*) human promoters, representatively. Error bars are represented as mean±S.D. ****P < 0.0001.


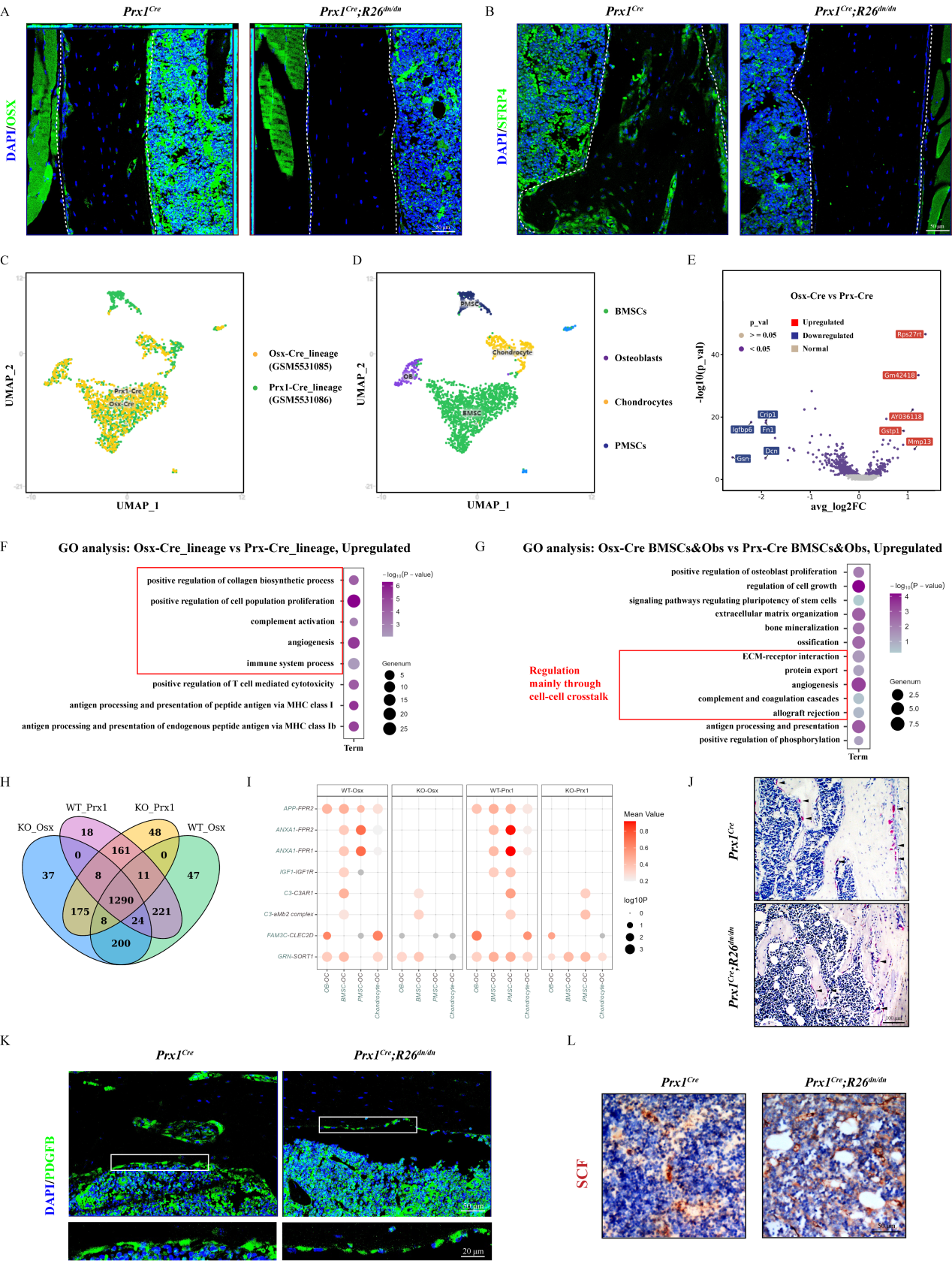


**Supplementary Figure 10.** (A) OSX expression in the femur bone of *Prx1^Cre^* and *Prx1^Cre^;R26^dn/dn^* mouse by IF staining. (B) SFRP4 expression in the femur bone of *Prx1^Cre^* and *Prx1^Cre^;R26^dn/dn^* mouse by IF staining. (C) Two previously reported scRNA-seq data, including femur Osx-Cre_lineage and femur Prx1-Cre_lineage, were visualized with UMAP. (D) Populations of BMSCs, osteoblasts, chondrocytes and PMSCs visualized as a UMAP plot. (E) Volcano plot displays global gene expression in the femur Osx-Cre_lineage cells and femur Prx1-Cre_lineage cells using scRNA-seq data. (F-G) The gene ontology (GO) terms are visualized in a bubble plot. (H) Cell communication analysis between Osx-Cre/Prx1-Cre lineage cells and osteoclasts & hematopoietic cells in *Osx^Cre^* and *Osx^Cre^;R26^dn/dn^* mice. (I) Cell communication analysis between Osx-Cre/Prx1-Cre lineage cells and osteoclasts & pre-osteoclasts in *Osx^Cre^* and *Osx^Cre^;R26^dn/dn^* mice. (J) TRAP staining of femur bone from 4-week-old *Prx1^Cre^;R26^dn/dn^* mice and their control littermates. (K) PDGFB expression in the femur bone of *Prx1^Cre^* and *Prx1^Cre^;R26^dn/dn^* mouse by IF staining. (L) SCF expression in the femur bone of *Prx1^Cre^* and *Prx1^Cre^;R26^dn/dn^* mouse by IHC staining.

**Table S1. Primers for RT-qPCR**

| **Gene** | **Primer_F** | **Primer_R** |
| --- | --- | --- |
| ***Hprt*** | GTTAAGCAGTACAGCCCCAAA | AGGGCATATCCAACAACAAACTT |
| ***βactin*** | ACCAACTGGGACGATATGGAGAAGA | TACGACCAGAGGCATACAGGGACAA |
| ***Runx2*** | CCAACCGAGTCATTTAAG | GCTCACGTCGCTCATCTTG |
| ***Bglap*** | CTTGGTGCACACCTAGCAGA | CTCCCTCATGTGTTGTCCCT |
| ***Col1a1*** | GCTCCTCTTAGGGGCCACT | CCACGTCTCACCATTGGGG |
| ***Osx*** | CCTTCCCTCACTCATTTCCTGG | TGTTGCCTGGACCTGGTGAGAT |
| ***Alp*** | CGGGACTGGTACTCGGATAA | ATTCCACGTCGGTTCTGTTC |
| ***Nfatc1*** | GACCCGGAGTTCGACTTCG | TGACACTAGGGGACACATAACTG |
| ***Ctsk*** | GAAGAAGACTCACCAGAAGCAG | TCCAGGTTATGGGCAGAGATT |
| ***Acp5*** | CACTCCCACCCTGAGATTTGT | CATCGTCTGCACGGTTCTG |
| ***Oscar*** | CCTAGCCTCATACCCCCAG | CGTTGATCCCAGGAGTCACAA |
| ***Dcstamp*** | GGGGACTTATGTGTTTCCACG | ACAAAGCAACAGACTCCCAAAT |
| ***Sfrp4*** | AGAAGGTCCATACAGTGGGAAG | GTTACTGCGACTGGTGCGA |
| ***Camp*** | GCTGTGGCGGTCACTATCAC | TGTCTAGGGACTGCTGGTTGA |
| ***C3*** | CCAGCTCCCCATTAGCTCTG | GCACTTGCCTCTTTAGGAAGTC |
| ***Vegfb*** | GCCAGACAGGGTTGCCATAC | GGAGTGGGATGGATGATGTCAG |
| ***Pdgfd*** | ATGCAACGGCTCGTTTTAGTC | CGGAGTCGCAAAAGTGTCC |
| ***Efna2*** | CGATACGCAGTCTACTGGAAC | GGTAGTCGTTGATGCTCACCT |
| ***Adipoq*** | TGTTCCTCTTAATCCTGCCCA | CCAACCTGCACAAGTTCCCTT |
| ***Sema4g*** | TCAGCTTTCTCACAGTAACAGC | CCCTTGAAGTGCCGAATCTGG |
| ***Sema4d*** | CCTGGTGGTAGTGTTGAGAAC | GCAAGGCCGAGTAGTTAAAGAT |

**Table S2 Antibody for flow cytometry analysis.**

| **antibody** | **manufacture** | **Catalog** |
| --- | --- | --- |
| IgD | BioLegend | 405713 |
| CD127 | BioLegend | 135011 |
| IgM | BioLegend | 406513 |
| CD43 | BioLegend | 143203 |
| CD135 | BioLegend | 135313 |
| CD34 | BioLegend | 128617 |
| Ly57 | BioLegend | 108307 |
| CD23 | BioLegend | 101630 |
| CD21/CD35 | BioLegend | 123409 |
| CD138 | BioLegend | 142523 |
| FcεRIα | BioLegend | 134319 |
| CD93 | BioLegend | 136509 |
| CD3 | BioLegend | 100314 |
| NK1.1 | BioLegend | 108739 |
| Ly6G | BioLegend | 127623 |
| CD24 | BioLegend | 101825 |
| CD19 | BioLegend | 115545 |

**Table S3. The Main Phenotype of Murine Models of Osteopetrosis**

| Mouse model | Studies | increased bone density and narrow marrow cavity | dwarfism | brittleness of bone | defect in bone resorption/number of osteoclasts | anemia and haemorrhagia | immune system disorders |
| --- | --- | --- | --- | --- | --- | --- | --- |
| *DnRARα^Osx^* | Our study | yes | yes | yes | yes/decrease | yes | yes |
| *Clcn7 -/-* | Ref 1,2 | yes | yes | yes | yes/decrease | yes | yes |
| *β3 -/-* | Ref 3,4 | yes | no | yes | yes/increase | yes | yes |
| *DAP -/-β3 -/-* | Ref 5 | yes | yes | yes | yes/normal | not reported | not reported |
| *Tcirg1-/-* | Ref 6,7 | yes | yes | yes | yes/normal or increase | not reported | not reported |
| *Ostm1-/-* | Ref 8 | yes | yes | yes | yes/increase | not reported | not reported |
| *op/op* | Ref 9 | yes | no | yes | yes/decrease | not reported | yes |
| *Rankl -/-* | Ref 10 | yes | yes | yes | yes/decrease | very mild | yes |
| *Csf1r -/-* | Ref 11,12 | yes | yes | yes | yes/decrease | yes | yes |
| *PU.1 -/-* | Ref 13 | yes | yes | yes | yes/decrease | yes | yes |
| *mi/mi* | Ref 14 | yes | yes | yes | yes/decrease | yes | yes |
| *Rank -/-* | Ref 15,16 | yes | yes | yes | yes/decrease | yes | yes |
| *c-fos -/-* | Ref 17 | yes | yes | yes | yes/decrease | not reported | yes |
| *Nfκb1/2 -/-* | Ref 18,19 | yes | yes | yes | yes/decrease | not reported | yes |
| *Traf6 -/-* | Ref 20,21 | yes | yes | yes | yes/decrease | not reported | yes |
| *c-src -/-* | Ref 22 | yes | yes | yes | yes/increase | not reported | not reported |
| *Ctsk -/-* | Ref 23,24 | yes | no | yes | yes/normal | yes | not reported |
| *Trap -/-* | Ref 25 | yes | yes | yes | yes/normal | not reported | not reported |

**Supplementary. Materials and Methods**

**Mice.** The *Rosa26-loxp-stop-loxp-dnRARα403* strain (*R26^dn/dn^*) was a gift from Prof. Cathy Mendelsohn (Columbia University, USA). The *Osterix*-Cre (*Osx^Cre^*) (No.006361) strain and *Prx1^Cre^* (No. 005584) strain were purchased from the Jackson Laboratory (Bar Harbor, ME, USA)^26^. The *Prrx1^CreERT2^* strain is a gift from Prof. Baojie Li (Shanghai Jiao Tong University, China). Both strains of mice were maintained on the C57BL/6 background. *R26^dn/dn^* mice were crossed with *Osx^Cre^* mice to generate *Osx^Cre^;R26^dn/dn^* mice. *R26^dn/dn^* mice were crossed with *Prx1^Cre^* mice to generate *Prx1^Cre^;R26^dn/dn^* mice. All mice were bred and maintained under specific pathogen free (SPF) conditions.

**Analysis of bone phenotype.** For alcian blue and ARS staining of the skeleton, briefly, newborn mice were eviscerated and the skin was removed. After fixation with 95% ethanol for 1 day, the newborns were stained for 42 hours in alcian blue solution. Then, they were fixed and cleared with 95% ethanol two times, each for 1 hour, and then treated with 2% KOH for 3–4 hours. After staining with ARS solution for 3–4 hours, the skeletons were cleared with 1% KOH in 20% glycerol. For 1-week-old mice, ARS staining required 6–8 hours, and then the skeletons were cleared with 1% KOH in 20% glycerol.

For histological analysis, bone tissues were fixed in 4% paraformaldehyde (PFA) for 48 hours, incubated in 15% DEPC-EDTA for decalcification, and then embedded in paraffin. For embryonic mice, 4 μm tissue sections were used for DIG-labelled *in situ* hybridization (No.11207733910; Roche, Basel, Switzerland) as described previously. For postnatal mice, bone tissues were fixed in 4% PFA and decalcified for 2 weeks prior to paraffin embedding. Tissue sections (6 μm) were used for H&E staining and tartrate-resistant acid phosphatase (TRAP) staining according to the standard protocol (387A-1KT; Sigma-Aldrich, St Louis, MO, USA) as described previously. Images were captured with a microscope (BX51, Olympus, Tokyo, Japan).

**Oil Red O staining.** 7-day-old mice were sacrificed with carbon dioxide and femurs were dissected and stored in 4% paraformaldehyde at 4°C for 24 hours, rinsed with PBS. After dehydration in 30% sucrose (in PBS) at 4°C overnight they were embedded in OCT (Sakura Finetek Co. Ltd., Tokyo, Japan) for preparation. Samples were cut into 10 μm-thick sections using a cryostat (HM525; Thermo Fisher Scientific, Waltham, MA, USA) and then were stored at -80°C. Cryosections were used for oil red O staining according to the standard protocol (C0157S, Beyotime). Images were captured with a microscope (BX51, Olympus, Tokyo, Japan).

**Immunohistochemical staining.** Dewaxed and rehydrated paraffin sections were subjected to antigen retrieval performed with citrate buffer solution (pH 6.0) at 95°C for 15 minutes. A solution of 3% H_2_O_2_ was used to block the activity of endogenous peroxidase. Sections were blocked with 3% BSA at room temperature for 30 min and then incubated with Perilipin-1 antibodies (CST, D1D8, Rabbit monoclonal, 1:1000) overnight at 4 °C and rinsed in PBS. Signals were developed using the DAB staining kit（Absin, abs957）.

**Calcein-alizarin red double labeling.** Mice received intraperitoneal injection of 20 mg/kg calcein (CA, 1 mg/mL in 2% NaHCO3 solution) on day 0, and 40 mg/kg Alizarin Red S (AL, 2 mg/mL in H_2_O) on day 4. Mice were sacrificed on day 7 and isolated tibiae were dehydrated and embedded in polymethylmethacrylate. Samples were cut into 5-µm sections with a hard tissue cutter (RM2265, Leica, Wetzlar, Germany) and fluorescence-labeled images were captured using a microscope (BX51, Olympus). The bone formation activity represented by mineral apposition rate (MAR) was measured according to a previously-described protocol.

**Micro-CT analysis.** Micro-CT analysis was performed according to our previous work. Mouse hind limbs were harvested, soft tissues were removed, and the remaining tissues were stored in 70% ethanol. The femora of 4-week-old and 12-week-old *Osx^Cre^*;*R26^dn/dn^* mice, *Osx^Cre^*;*R26^dn/-^* mice and control littermates were collected for micro-CT scanning with a 10-μm voxel size. The femora of 4-week-old *Prx1^Cre^*;*R26^dn/dn^* mice, *Prx1^Cre^*;*R26^dn/-^* mice and control littermates were collected for micro-CT scanning with a 9-μm voxel size (Skyscan1172). One hundred slices were selected for micro-CT scanning, trabecular microarchitecture parameters including bone volume fraction (BV/TV), trabecular thickness (Tb.Th.) and trabecular number (Tb.N.) were measured following the introductions of the manufacturer. Fifty slices from the middle of the femora were used to analyze cortical thickness (Ct.Th.).

**Three-point bending test.** Femora from 4-week-old mice were collected and stored in 75% ethanol. Three-point bending tests were performed using an Instron 3345 universal testing machine (Instron, Canton, MA, USA) at the femora midshaft with a displacement rate of 0.03 mm/s until the bone fractured. Maximum load was determined using load-deflection diagrams.

**Cell culture.** Bone marrow stem cells (BMSCs) were isolated from the femora and tibiae of 4-week-old mice and cultured in Minimum Essential Medium-α (α-MEM, 10-022-CVR; Corning Life Sciences, Corning, NY, USA) containing 10% FBS (WS500T; Ausbian) and 1% Penicillin/Streptomycin (No.15140122; Thermo Fisher Scientific, Waltham, MA, USA). Half of the culture medium was changed after 3 days of culture, and all the culture medium was replaced after 5 days, then the cells were counted and re-plated on the 7th day of culture. For *in vitro* osteoblast differentiation, 2×10^5^ BMSCs were seeded into wells of 12-well plates and cultured in osteogenic medium (MUBMD-90021; Cyagen Biosciences Inc., Santa Clara, CA, USA) and subjected to alkaline phosphatase (ALP) staining (P0321S; Beyotime Institute of Biotechnology, Jiangsu, China) on day 7 and ARS staining (MUBMD-90021; Cyagen) on day 14. For the osteoblast and osteoclast co-culture system, the mouse macrophage cell line (Raw 264.7) was obtained from American Type Culture Collection (ATCC; Manassas, VA, USA). After BMSC-derived osteoblastic cells were seeded in culture plates for 24 h, the Raw 264.7 cells were seeded at a density of 2×10^4^ cells/mL in the presence of 1, 25-dihydroxyvitamin D3 (10 nM) (MCE, Monmouth Junction, NJ, USA) and PGE2 (1 μM) (MCE) diluted with α-MEM. The culture medium was changed every 48 hours. TRAP staining was conducted 5 days after co-culture initiation.^27^ The cells were cultured in a 5% CO_2_ humidified incubator at 37°C. 1 μg/mL Complement C3 recombinant protein (Abclonal, RP01415) was used for intervention according to a previous research^28^. For cells digested from femur bone without bone marrow (F_dig cells), bone marrow was flushed out and bone were first washed with phosphate-buffered saline (PBS), cut into small pieces (approximately 1mm^3^) on ice and enzymatically digested with collagenase/dispase for 35 min at 37 °C. The digested supernatant was moved into α-MEM for neutralization and centrifuged at 400 g for 5 min to collect the digested cells for culture. For cells digested from parietal bone (P_dig cells), parietal bones were first washed with phosphate-buffered saline (PBS), cut into small pieces (approximately 1mm^3^) on ice and enzymatically digested with collagenase/dispase for 35 min at 37 °C. The digested supernatant was moved into α-MEM for neutralization and centrifuged at 400 g for 5 min to collect the digested cells for culture.

**Adenoviruses infection.** Ad-eGFP and Ad-Cre adenovirus were purchased from Hanbio Biotechnology Co. Ltd (Shanghai). 2×10^5^ cells were seeded into wells of 12-well plates in α-MEM for 24 hours, then cells were infected with adenovirus expressing either Ad-eGFP or Ad-Cre at a moi of 100. After 6 hours, cells were cultured or collected for further studies. 0.1μg/mL SFRP4 recombinant protein (R&D, 9806-SF) was used for intervention.

**RT-PCR analysis.** Total RNA was extracted from cells using TRIzol and was reverse-transcribed into cDNA using the Prime Script RT master kit (RR036A; TakaRa Bio Inc., Shiga, Japan). Real-time reverse transcription PCR (RT-PCR) was performed using the Bio-Rad CFX96 system. The primer used were listed in Table S1.

**Luciferase reporter assay.** We assessed *Sfrp4, Camp, Pdgfd* or *Sema4d* transcriptional activity using the *Sfrp4, Camp, Pdgfd* or *Sema4d-*responsive dual-luciferase reporter assay system. 293T cells were plated at a density of 2×10^5^ cells/well in a 24-well plate for 18 hours and transfected using Lipofectamine 8000 (C0533; Beyotime) leveraging luciferase reporter plasmid (*Sfrp4-luc, Camp-luc, Pdgfd-luc* or *Sema4d-luc*) and Renilla along with RARα. All wells were supplemented with control empty expression vector plasmids to keep the total amount of DNA constant. At 48 hours after transfection, luciferase activity was assayed in cell extract supernatants using the Dual-Luciferase Reporter Assay System according to the manufacturer’s protocol (E1910; Promega, Madison, WI, USA).

**Flow cytometry analysis.** Dead cells were stained with fixable viability stain 520 (BD Biosciences) and removed. CD16/32 antibody (Biolegend) was used to block nonspecific binding to Fc receptors before surface staining. For surface staining, cells were resuspended in 100 μL of FACS buffer (BD Biosciences) containing antibody cocktails and stained at 4 °C in the dark for 30minutes. Antibodies used for flow cytometry analysis are listed in Supplementary Table 1. All flow data were acquired by BD FACSDiva software v8.0.2 and analyzed by FlowJo VX.

**Hematology analyze.** Peripheral blood of mice was derived and analyzed using Mindray BC-500.

**Single-cell Dissociation.** 3 mice for each group (*Osx^Cre^* for WT group, *Osx^Cre^;R26^dn/dn^* for KO group) and both side of the femur were collected. For bone groups, bone marrow was flushed out and bone were first washed with phosphate-buffered saline (PBS), cut into small pieces (approximately 1mm^3^) on ice and enzymatically digested with collagenase/dispase for 35 min at 37 °C according to our previous study. After digestion, samples were sieved through a 40µm cell strainer, and centrifuged at 300g for 5 min. After the supernatant was removed, the pelleted cells were suspended in red blood cell lysis buffer (Miltenyi Biotec) to lyse red blood cells. After washing with PBS containing 0.04% BSA, the cell pellets were re-suspended in PBS containing 0.04% BSA and re-filtered through a 35μm cell strainer. Dissociated single cells were then stained with AO/PI for viability assessment using Countstar Fluorescence Cell Analyzer.

For bone marrow group, bone marrow was flushed out and sieved through a 40µm cell strainer, and centrifuged at 300g for 5 min. The following steps are same to the bone group.

**Single-cell Sequencing.** The scRNA-Seq libraries were generated using the 10X Genomics Chromium Controller Instrument and Chromium Single Cell 3’V3.1 Reagent Kits (10X Genomics, Pleasanton, CA). Briefly, cells were concentrated to approximately 1000 cells/uL and loaded into each channel to generate single-cell Gel Bead-In-Emulsions (GEMs). After the RT step, GEMs were broken and barcoded-cDNA was purified and amplified. The amplified barcoded cDNA was fragmented, A-tailed, ligated with adaptors and index PCR amplified. The final libraries were quantified using the Qubit High Sensitivity DNA assay (Thermo Fisher Scientific) and the size distribution of the libraries were determined using a High Sensitivity DNA chip on a Bioanalyzer 2200 (Agilent). All libraries were sequenced by illumina sequencer (Illumina, San Diego, CA) on a 150 bp paired-end run.

**Single-cell RNA Statistical Analysis.** ScRNA-seq data analysis was performed by NovelBio Co.,Ltd. with NovelBrain Cloud Analysis Platform (www.novelbrain.com). We applied fastp with default parameter filtering the adaptor sequence and removed the low quality reads to achieve the clean data^29^. Then the feature-barcode matrices were obtained by aligning reads to the mouse genome (mm10, Ensembl 100) using CellRanger v7.1.0. We applied the down sample analysis among samples sequenced according to the mapped barcoded reads per cell of each sample and finally achieved the aggregated matrix. Cells contained over 200 expressed genes and mitochondria UMI rate below 10% passed the cell quality filtering and mitochondria genes were removed in the expression table.

Seurat package (version: 4.1.1, https://satijalab.org/seurat/) was used for cell normalization and regression based on the expression table according to the UMI counts of each sample and percent of mitochondria rate to obtain the scaled data. PCA was constructed based on the scaled data with top 2000 high variable genes and top 10 principals were used for UMAP construction. Utilizing graph-based cluster method, we acquired the unsupervised cell cluster result based the PCA top 10 principal and we calculated the marker genes by FindAllMarkers function with wilcox rank sum test algorithm under following criteria:1. Log2FC > 0.25; 2. Pvalue < 0.05; 3. min.pct > 0.1. In order to identify the cell type detailed, the clusters of same cell type were selected for re-UMAP analysis, graph-based clustering and marker analysis.

Two previously reported scRNA-seq data sets (GSM5531085 and GSM5531086) were retrieved to explore the differences between femur Osx-Cre lineage cells and femur Prx-Cre lineage cells. The upregulated genes in Osx-Cre set were screened for GO analysis.

**Cell Communication Analysis.** To enable a systematic analysis of cell-cell communication molecules, we applied cell communication analysis based on the CellPhoneDB^30^, a public repository of ligands, receptors and their interactions. Membrane, secreted and peripheral proteins of the cluster of different time point was annotated. Significant mean and Cell Communication significance (p-value<0.05) was calculated based on the interaction and the normalized cell matrix achieved by Seurat Normalization.

**RNA-Seq and GO analysis.** BMSCs were isolated from the femur of 4-week-old *Osx^Cre^* mice (n=3) and *Osx^Cre^*;*R26^dn/dn^* mice (n=3) littermates and cultured 7 days and cultured in osteogenic medium for another 7 days as described above for osteoblasts. Total RNA was isolated using TRIzol (T9424; Sigma). Complementary DNA library preparation and sequencing were performed according to the Illumina standard protocol. Raw reads were mapped to mm9 using the TopHat version 1.4.1 program. We assigned fragment per kilo base per million (FPKM) as an expression value for each gene using Cufflinks version 1.3.0 software. Then, Cuffdiff software was used to identify differentially-expressed genes between *Osx^Cre^*;*R26^dn/dn^* and control *Osx^Cre^* samples. Differentially-expressed gene heat maps were clustered by k-means clustering using the Euclidean distance as the distance and visualized using Java TreeView software. GO analysis was performed with the DAVID online tool. Top GO categories were selected according to the *P* values.

**Reference**

1 Sobacchi, C., Villa, A., Schulz, A. & Kornak, U. in *GeneReviews(®)* (eds M. P. Adam *et al.*) (University of Washington, Seattle

Copyright © 1993-2023, University of Washington, Seattle. GeneReviews is a registered trademark of the University of Washington, Seattle. All rights reserved., 1993).

2 Kornak, U., Kasper, D., Bösl, M., Kaiser, E., Schweizer, M., Schulz, A. *et al.* Loss of the ClC-7 chloride channel leads to osteopetrosis in mice and man. *Cell* **104**, 205-215 (2001).

3 McHugh, K., Hodivala-Dilke, K., Zheng, M., Namba, N., Lam, J., Novack, D. *et al.* Mice lacking beta3 integrins are osteosclerotic because of dysfunctional osteoclasts. *The Journal of clinical investigation* **105**, 433-440 (2000).

4 Moser, M., Nieswandt, B., Ussar, S., Pozgajova, M. & Fässler, R. Kindlin-3 is essential for integrin activation and platelet aggregation. *Nature medicine* **14**, 325-330 (2008).

5 Zou, W. & Teitelbaum, S. Absence of Dap12 and the αvβ3 integrin causes severe osteopetrosis. *The Journal of cell biology* **208**, 125-136 (2015).

6 Capo, V., Abinun, M. & Villa, A. Osteoclast rich osteopetrosis due to defects in the TCIRG1 gene. *Bone* **165**, 116519 (2022).

7 Li, Y., Chen, W., Liang, Y., Li, E. & Stashenko, P. Atp6i-deficient mice exhibit severe osteopetrosis due to loss of osteoclast-mediated extracellular acidification. *Nature genetics* **23**, 447-451 (1999).

8 Rajapurohitam, V., Chalhoub, N., Benachenhou, N., Neff, L., Baron, R. & Vacher, J. The mouse osteopetrotic grey-lethal mutation induces a defect in osteoclast maturation/function. *Bone* **28**, 513-523 (2001).

9 Naito, M., Umeda, S., Takahashi, K. & Shultz, L. Macrophage differentiation and granulomatous inflammation in osteopetrotic mice (op/op) defective in the production of CSF-1. *Molecular reproduction and development* **46**, 85-91 (1997).

10 Kong, Y., Yoshida, H., Sarosi, I., Tan, H., Timms, E., Capparelli, C. *et al.* OPGL is a key regulator of osteoclastogenesis, lymphocyte development and lymph-node organogenesis. *Nature* **397**, 315-323 (1999).

11 Keshvari, S., Caruso, M., Teakle, N., Batoon, L., Sehgal, A., Patkar, O. *et al.* CSF1R-dependent macrophages control postnatal somatic growth and organ maturation. *PLoS genetics* **17**, e1009605 (2021).

12 Li, J., Chen, K., Zhu, L. & Pollard, J. Conditional deletion of the colony stimulating factor-1 receptor (c-fms proto-oncogene) in mice. *Genesis (New York, N.Y. : 2000)* **44**, 328-335 (2006).

13 Tondravi, M., McKercher, S., Anderson, K., Erdmann, J., Quiroz, M., Maki, R. *et al.* Osteopetrosis in mice lacking haematopoietic transcription factor PU.1. *Nature* **386**, 81-84 (1997).

14 Weilbaecher, K., Motyckova, G., Huber, W., Takemoto, C., Hemesath, T., Xu, Y. *et al.* Linkage of M-CSF signaling to Mitf, TFE3, and the osteoclast defect in Mitf(mi/mi) mice. *Molecular cell* **8**, 749-758 (2001).

15 Dougall, W., Glaccum, M., Charrier, K., Rohrbach, K., Brasel, K., De Smedt, T. *et al.* RANK is essential for osteoclast and lymph node development. *Genes & development* **13**, 2412-2424 (1999).

16 Li, J., Sarosi, I., Yan, X., Morony, S., Capparelli, C., Tan, H. *et al.* RANK is the intrinsic hematopoietic cell surface receptor that controls osteoclastogenesis and regulation of bone mass and calcium metabolism. *Proc. Natl. Acad. Sci. U. S. A.* **97**, 1566-1571 (2000).

17 Wang, Z., Ovitt, C., Grigoriadis, A., Möhle-Steinlein, U., Rüther, U. & Wagner, E. Bone and haematopoietic defects in mice lacking c-fos. *Nature* **360**, 741-745 (1992).

18 Xing, L., Chen, D. & Boyce, B. Mice Deficient in NF-κB p50 and p52 or RANK Have Defective Growth Plate Formation and Post-natal Dwarfism. *Bone research* **1**, 336-345 (2013).

19 Iotsova, V., Caamaño, J., Loy, J., Yang, Y., Lewin, A. & Bravo, R. Osteopetrosis in mice lacking NF-kappaB1 and NF-kappaB2. *Nature medicine* **3**, 1285-1289 (1997).

20 Naito, A., Azuma, S., Tanaka, S., Miyazaki, T., Takaki, S., Takatsu, K. *et al.* Severe osteopetrosis, defective interleukin-1 signalling and lymph node organogenesis in TRAF6-deficient mice. *Genes to cells : devoted to molecular & cellular mechanisms* **4**, 353-362 (1999).

21 Lomaga, M., Yeh, W., Sarosi, I., Duncan, G., Furlonger, C., Ho, A. *et al.* TRAF6 deficiency results in osteopetrosis and defective interleukin-1, CD40, and LPS signaling. *Genes & development* **13**, 1015-1024 (1999).

22 Soriano, P., Montgomery, C., Geske, R. & Bradley, A. Targeted disruption of the c-src proto-oncogene leads to osteopetrosis in mice. *Cell* **64**, 693-702 (1991).

23 Pennypacker, B., Shea, M., Liu, Q., Masarachia, P., Saftig, P., Rodan, S. *et al.* Bone density, strength, and formation in adult cathepsin K (-/-) mice. *Bone* **44**, 199-207 (2009).

24 Jacome-Galarza, C., Soung, d. Y., Adapala, N., Pickarski, M., Sanjay, A., Duong, L. *et al.* Altered hematopoietic stem cell and osteoclast precursor frequency in cathepsin K null mice. *Journal of cellular biochemistry* **115**, 1449-1457 (2014).

25 Hollberg, K., Hultenby, K., Hayman, A., Cox, T. & Andersson, G. Osteoclasts from mice deficient in tartrate-resistant acid phosphatase have altered ruffled borders and disturbed intracellular vesicular transport. *Experimental cell research* **279**, 227-238 (2002).

26 Zhou, S., Dai, Q., Huang, X., Jin, A., Yang, Y., Gong, X. *et al.* STAT3 is critical for skeletal development and bone homeostasis by regulating osteogenesis. *Nature communications* **12**, 6891 (2021).

27 Dai, Q., Han, Y., Xie, F., Ma, X., Xu, Z., Liu, X. *et al.* A RANKL-based Osteoclast Culture Assay of Mouse Bone Marrow to Investigate the Role of mTORC1 in Osteoclast Formation. *Journal of visualized experiments : JoVE* (2018).

28 Ignatius, A., Schoengraf, P., Kreja, L., Liedert, A., Recknagel, S., Kandert, S. *et al.* Complement C3a and C5a modulate osteoclast formation and inflammatory response of osteoblasts in synergism with IL-1β. *Journal of cellular biochemistry* **112**, 2594-2605 (2011).

29 Chen, S., Zhou, Y., Chen, Y. & Gu, J. fastp: an ultra-fast all-in-one FASTQ preprocessor. *Bioinformatics (Oxford, England)* **34**, i884-i890 (2018).

30 Vento-Tormo, R., Efremova, M., Botting, R., Turco, M., Vento-Tormo, M., Meyer, K. *et al.* Single-cell reconstruction of the early maternal-fetal interface in humans. *Nature* **563**, 347-353 (2018).
